# Hydrophilic fluorinated molecules for spectral ^19^F MRI

**DOI:** 10.1038/s41598-018-21178-3

**Published:** 2018-02-13

**Authors:** Eric A. Tanifum, Chandreshkumar Patel, Matthew E. Liaw, Robia G. Pautler, Ananth V. Annapragada

**Affiliations:** 1 Department of Pediatric Radiology, Texas Children’s Hospital, Houston, TX 77030 USA; 2 Department of Radiology, Baylor College of Medicine, Houston, TX 77030 USA; 3 School of Medicine, Baylor College of Medicine, Houston, TX 77030 USA; 4 Department of Molecular Physiology and Biophysics, Baylor College of Medicine, Houston, TX 77030 USA; 5 Department of Biomedical Engineering, UT Southwestern Medical Center, Dallas, TX USA

## Abstract

Fluorine-19 (^19^F) Magnetic Resonance Imaging (MRI) is an emerging modality for molecular imaging and cell tracking. The hydrophobicity of current exogenous probes, perfluorocarbons (PFCs) and perfluoropolyethers (PFPEs), limits the formulation options available for *in vivo* applications. Hydrophilic probes permit more formulation flexibility. Further, the broad Nuclear Magnetic Resonance (NMR) chemical shift range of organofluorine species enables multiple probes with unique ^19^F MR signatures for simultaneous interrogation of distinct molecular targets *in vivo*. We report herein a flexible approach to stable liposomal formulations of hydrophilic fluorinated molecules (each bearing numerous magnetically equivalent ^19^F atoms), with ^19^F encapsulation of up to 22.7 mg/mL and a per particle load of 3.6 × 10^6 19^F atoms. Using a combination of such probes, we demonstrate, with no chemical shift artifacts, the simultaneous imaging of multiple targets within a given target volume by spectral ^19^F MRI.

## Introduction

There has been a surge in interest in ^19^F MRI as a molecular imaging and cell tracking modality, driven in part by advances in MR technology including improvement in radiofrequency (RF) coil design, the development of dual ^19^F/^1^H imaging, and advanced scan protocols^1^. The gyromagnetic ratio of fluorine (40.08 MHz^.^T^−1^) is very close to that of the proton (42.58 MHz^.^T^−1^). So, current ^1^H MRI instruments require minimal hardware upgrades to acquire ^19^F-based images^1,2^. Further, there is no MRI-detectable endogenous ^19^F in soft tissue, leading to images with very high signal-to-noise ratio (SNR). The signal intensity from a ^19^F MR image unlike that from a conventional ^1^H MR T_1_ contrast agent such as Gadolinium, is directly proportional to contrast concentration, allowing direct and unambiguous quantification of disease activity^3,4^.

In spite of its potential to revolutionize molecular imaging, success with this modality has been primarily in the area of cell tracking. This can be attributed in part to challenges in the design and formulation of new probes suitable for other *in vivo* applications. For instance, a large number of ^l9^F atoms (generally high micromolar to milimolar concentrations)^1^, are required in each voxel of the target volume to enable high conspicuity in ^19^F MRI. Different approaches have been explored to address this limitation. The design and clinical applications of the most advanced amongst them (including emulsions of PFCs and PFPEs, hyperfluorinated molecules such as PERFECTA, fluorodendrimers and perfluorinated amphiphiles), were recently reviewed by Tirotta *et al*.^5^. Based on the review, PFC and PFPE dominate the preclinical/clinical space. This can be expected given the high percentage ^19^F atoms in these molecules. However, they are highly hydrophobic, limiting formulation to water emulsions. These emulsions generally require the extensive use of surfactants, have limited shelf stability, low biocompatibility and degradability^6^. Furthermore, most of these molecules have magnetically diverse fluorine atoms resulting in chemical shift artifacts and diffuse ^19^F MR images^7^.

Organofluorine molecules have a chemical shift range of >350 ppm^5^ and a novel technique introduced by Goette *et al*.^8^ showed that an excitation bandwidth of just 1–2 kHz is adequate to separately image complex ^19^F spin systems with high SNR. These suggest a theoretical possibility of developing a series of as many as 30 organofluorine probes with unique MR signatures. From this, one can envision interrogating different aspects of the heterogeneous pathological makeup of diseases (such as cancer^9^ or Alzheimer’s disease^10^), in a single imaging session by administering a cocktail of such probes targeted to each aspect of the pathology. A readout on the location and concentration of each probe can then be obtained by performing ^19^F MRI scans with the excitation pulse centered at its resonance frequency on the ^19^F NMR spectrum of the mixture.

The liposome nanoparticle platform, with the capacity to carry >1,000,000 hydrophilic fluorinated molecules in the aqueous interior of each particle, is well suited to test this hypothesis because of its intrinsic advantage as a nanovector^11^. As proof-of-concept, we designed, synthesized, and characterized four hydrophilic non-ionic fluorinated molecules **ET0863**, **ET0876**, **ET0886**, and **ET090**, each bearing magnetically equivalent ^19^F atoms. These were formulated into stable liposomes and tested as a new class of contrast agents for spectral ^19^F MRI. We demonstrated, using *in vitro* phantoms as well as subcutaneous and intramuscular deposits of these formulations that they can be selectively imaged within a target volume even in the presence of the widely used inhalable fluorinated anesthetic, isoflurane, without any interference or chemical shift artifacts.

## Results

**Figure 1:**
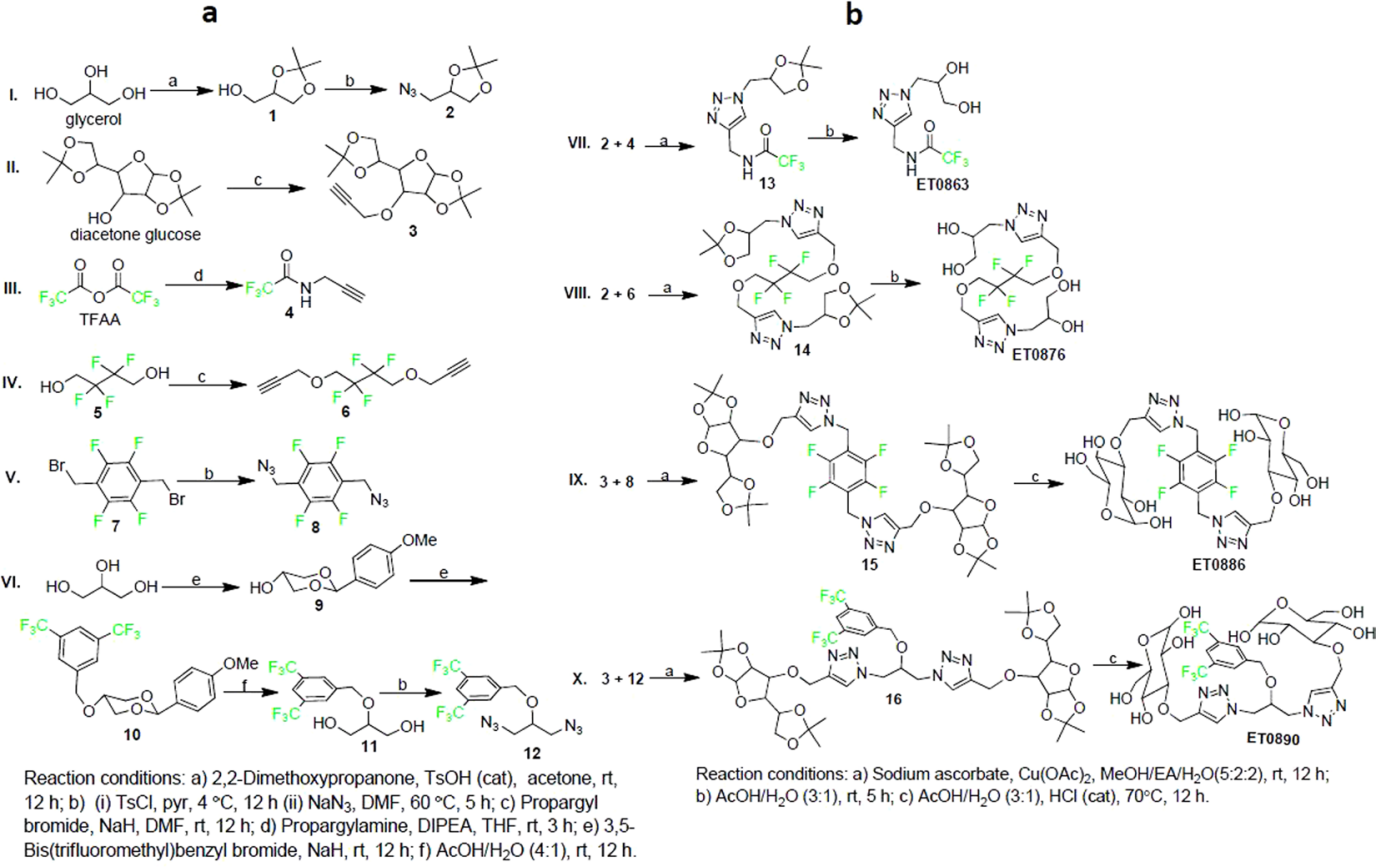
Synthetic routes to intermediates and the final molecules. (**a**) Synthesis of precursors of hydrophilic and fluorinated moieties. (**b**) “Click” reactions to couple fragments and subsequent deprotection to obtain desired hydrophilic fluorinated molecules.

The molecular design employed fragments of two non-ionic hydrophilic molecules (glycerol and glucose), and ‘click’ chemistry^12^, as the key synthetic step to couple them to the fluorinated species. The basic requirement for the fluorinated moiety was to have magnetically equivalent ^19^F atoms (in order to have a single NMR resonance frequency) per molecule to generate a strong and sharp ^19^F MRI signal. Preliminary evaluation of some commercially available organofluorine starting materials at 9.4 T, suggested a minimum peak separation of ~12 ppm between the resonance frequencies of individual species, to generate an image of each without any chemical shift artifacts. The 3,5-bis(trifluoro)phenyl-, trifluoroacetyl-, 1,2-bis(difluoro)methylene-, and disubstituted 2,3,5,6-tetrafluorophenyl- groups showed adequate resonance frequency separation and were selected for the study. Precursors of both the fluorinated and hydrophilic moieties were introduced in the late stage of the synthesis as preformed scaffolds (Fig. 1a**)** of either the azido or alkynyl derivative. Coupling of the respective hydrophilic and fluorinated moieties followed by deprotection to obtain the final compounds (Fig. 1b) all proceeded with excellent yields, and were optimized to generate gram quantities of each compound. All intermediates and final compounds were characterized by ^1^H and ^13^C NMR, HRMS, and ^19^F NMR (where applicable).

**Figure 2:**
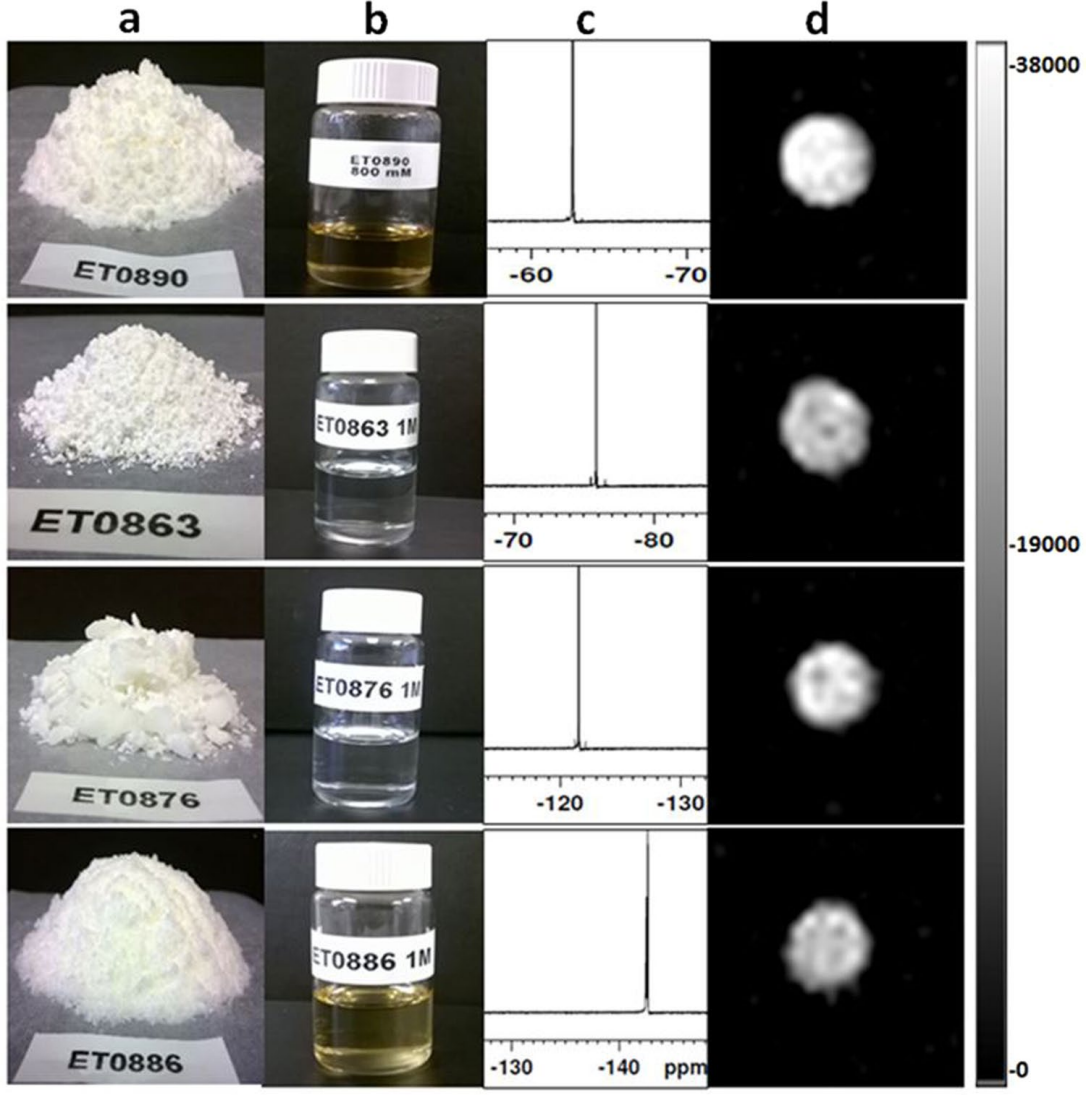
Physical and magnetic resonance properties of compounds. (**a**) All compounds are obtained as solids at room temperature. (**b**) All compounds dissolve in aqueous media to give clear solutions suitable for liposome formulation at room temperature. (**c**) ^19^F NMR spectra of each compound gives a single peak indicative of its magnetic and chemical purity. (**d**) ^19^F MRI obtained with a MSME scan protocol (Excitation bandwidth = 2000 Hz, TR = 2000 ms, TE = 8.95 ms, scan time = 10 min 40 s) of phantoms show single sharp image for each solution.

As shown in Fig. 2a, all final products were obtained as solids at room temperature and readily dissolved in aqueous media (water, saline, PBS, and histidine/saline buffers), to give clear solutions (Fig. 2b). ^19^F NMR of the solutions each gave a single peak: −75.90, −121.57, −143.39 and −62.65 ppm for **ET0863**, **ET0876**, **ET0886** and **ET0890** respectively (Fig. 2c). The single peaks indicate both the magnetic and chemical purity of each compound (estimated overall chemical purity >95%). ^19^F MR images of phantoms of these solutions showed no chemical shift artifacts as expected (Fig. 2d).

Stability testing in plasma at 37 °C showed that **ET0863** liposomes leaked more than 5% of their content within a 1.5 hour test period, and were excluded from subsequent experiments. **ET0876**, **ET0886**, and **ET0890** liposomes all showed less than 5% leak, and were retained for subsequent experiments. Table 1 shows the mean particle size, total lipid concentration, and the ^19^F content of each formulation. Assuming a lipid bilayer thickness of 4 nm, the total particles/mL and the total ^19^F atoms per particle were also estimated for each formulation based on the total encapsulated volume.

**Table 1:** Characteriztion of liposome formulations.

Liposome Formulation	Mean Particle Diameter (nm)	Polydispersity Index (PDI)	[Total Lipid] (mM)	[^19^F] (mg/mL)	Est. Number of Particles/mL	^19^F Atoms/Particle	Plasma Leak
**ET0876**	164.8 ± 1.2	0.10 ± 0.04	142.7 ± 1.5	22.7 ± 1.4	2.0 × 10^14^	3.6 × 10^6^	<5%
**ET0886**	166.7 ± 3.6	0.08 ± 0.07	142.6 ± 1.7	21.9 ± 0.3	2.0 × 10^14^	3.5 × 10^6^	<5%
**ET0890**	166.3 ± 7.1	0.10 ± 0.04	133.1 ± 1.0	16.6 ± 1.7	1.9 × 10^14^	1.4 × 10^6^	<5%

Data are represented as mean value ± SD.

**Figure 3:**
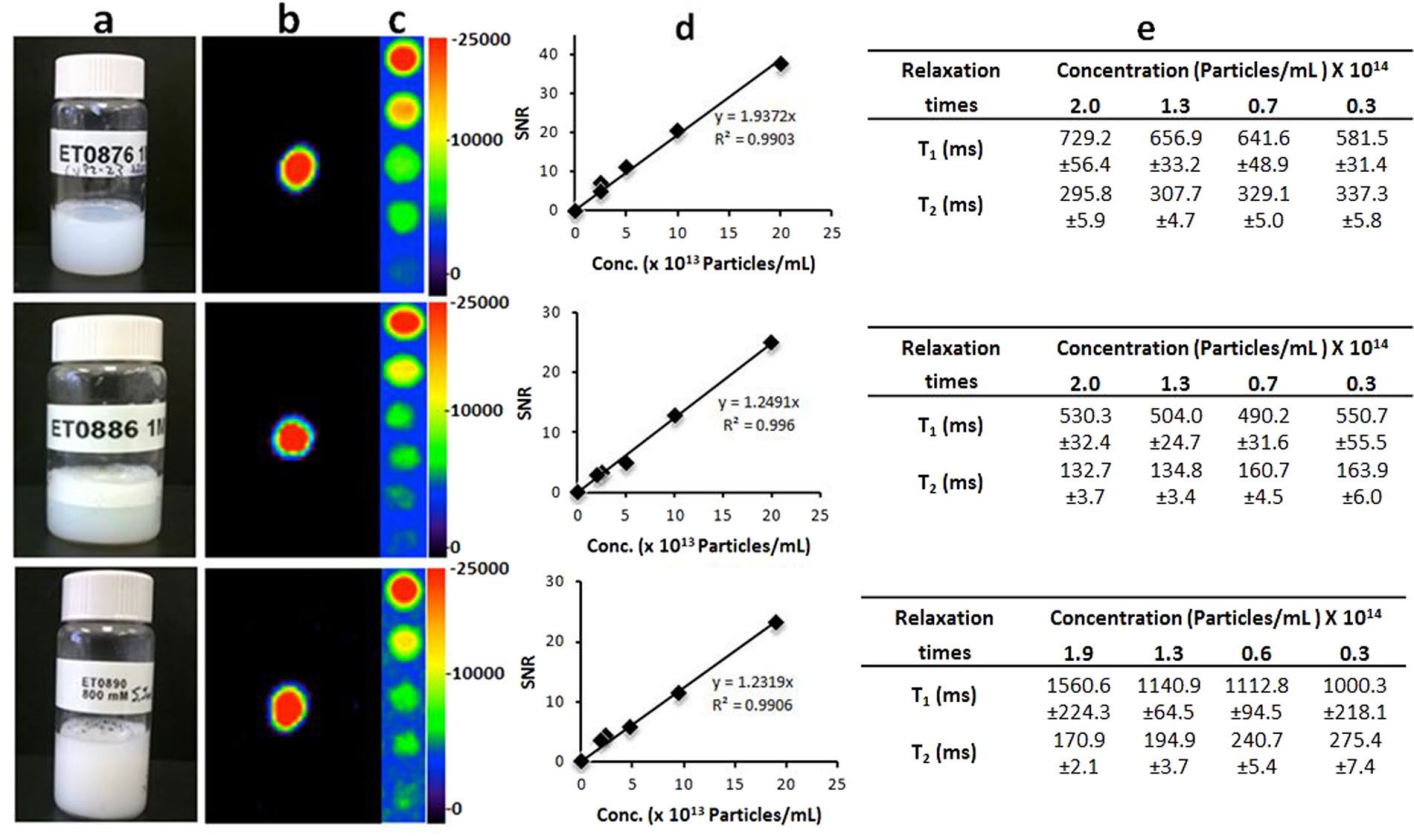
Properties of liposome formulations (**a**) Stable formulations of particles with low polydispersity. (**b**) Pseudocolored ^19^F MRI signal of phanthoms from each formulation obtained with a ^19^F MSME (Excitation bandwidth = 2000 Hz, TR = 2000 ms, TE = 8.95 ms, scan time = 10 min 40 s) scan protocol. (**c**) Dilution studies on each formulation suggest a signal with SNR > 4 can be obtained from each at over 5 fold dilution. (**d**) Plot of SNR against concentration shows linearity. (**e**) T_1_ and T_2_ relaxation times for neat formulations and dilutions thereof. Data are represented as mean value ± SD.

^19^F MRI scans were perfomed on phantoms of each neat formulation (Fig. 3a), using similar scan parameters as in the solution phantoms (data rendered in pseudo-color, Fig. 3b). ^19^F signal was recorded, with Signal to Noise Ratio (SNR) of 38, 25, and 23 for **ET0876**, **ET0886**, and **ET0890** formulations respectively. Dilution studies (Fig. 3c), showed that solutions of each formulation with as low as 2.5 × 10^13^ particles/mL were detectable by ^19^F MRI. A plot of the SNR against particle concentration (Fig. 3d), exhibited a linear relationship for each formulation. Relaxation times T_1_ and T_2_ (Fig. 3e) were determined for each neat formulation and diluted samples (using similar scan parameters as reported by Tirotta *et al*.^13^).

**Figure 4:**
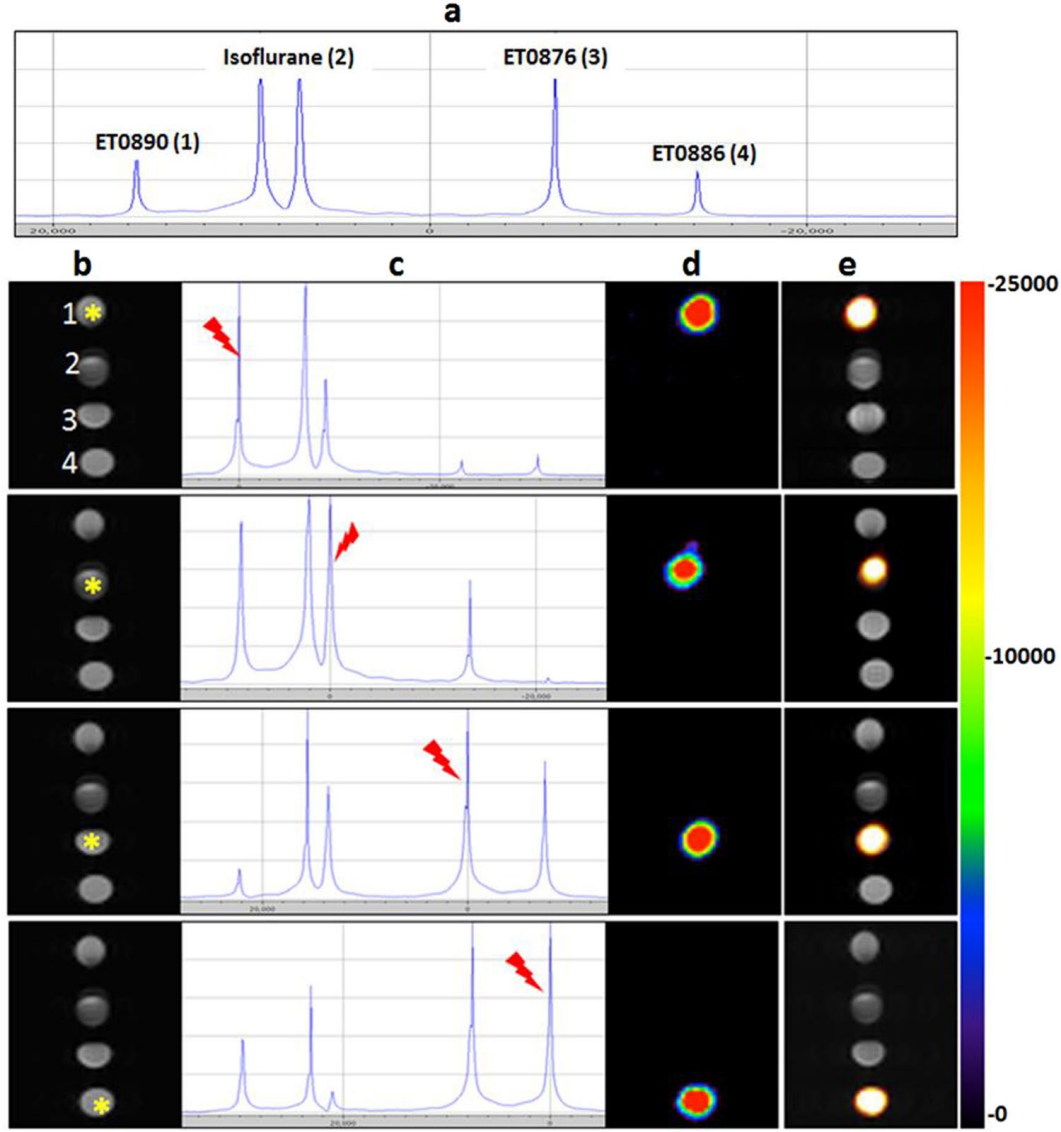
*In vitro* evaluation of ^19^F MRI spectral imaging potential of probes. (**a**) Single pulse ^19^F MR scan of a set of phantoms comprising the three formulations and Isofluran show resonance frequencies of all fluorine species in the magnet. (**b**) TurboRARE T_2_ (TR = 2500 ms, TE = 11 ms, Rare factor = 4, scan time = 5 min 20 s, Matrix size = 64 × 64), ^1^H MR image of phantoms (1 = **ET0890**, 2 = Isoflurane, 3 = **ET0876**, and 4 = **ET0886**), mimicking anatomy of subject in the bore of the magnet. (**c**) Single pulse ^19^F MR scan with resonance frequency of the starred phantom in (**a**), indicated by the red pointer. (**d**) ^19^F MR image following a ^19^F MSME scan (same scan parameters as in Fig. 3 above), of the selected frequency. The artifact observed in phantom number 2 (Isoflurane) is due to the second Isoflurane peak. (**e**) Overlay of ^19^F MR image over ^1^H MR image of all the phantoms, reports on the exact location of each phantom on the rack.

To evaluate the potential of these formulations as probes for spectral ^19^F MRI, phantoms of all three were scanned together with a phantom containing the fluorinated inhalable anesthetic, Isoflurane. A single pulse ^19^F MR resonance frequency sweep (Fig. 4a), showed peaks corresponding to all the fluorine species in the phantoms: **ET0890** (one peak), Isoflurane (two peaks), **ET0876** (one peak), and **ET0886** (one peak). An image from a ^1^H T_2_ Rapid Acquisition with Relaxation Enhancement (RARE) scan, representative of an anatomy scan (Fig. 4b), showed the position of each phantom within the bore of the magnet: **ET0890** (position 1), Isoflurane (position 2), **ET0876** (position 3), and **ET0886** (position 4). When the phantoms were subjected to the same ^19^F MSME scan sequence as above, with the excitation bandwith centered at the highlighted peak position (Fig. 4c), only that phantom generated a visible ^19^F MR image (shown in pseudo-color, Fig. 4d). An overlay of the ^19^F image over the ^1^H image allowed visualization of the exact location of that signal in the entire field of view (Fig. 4e).

**Figure 5:**
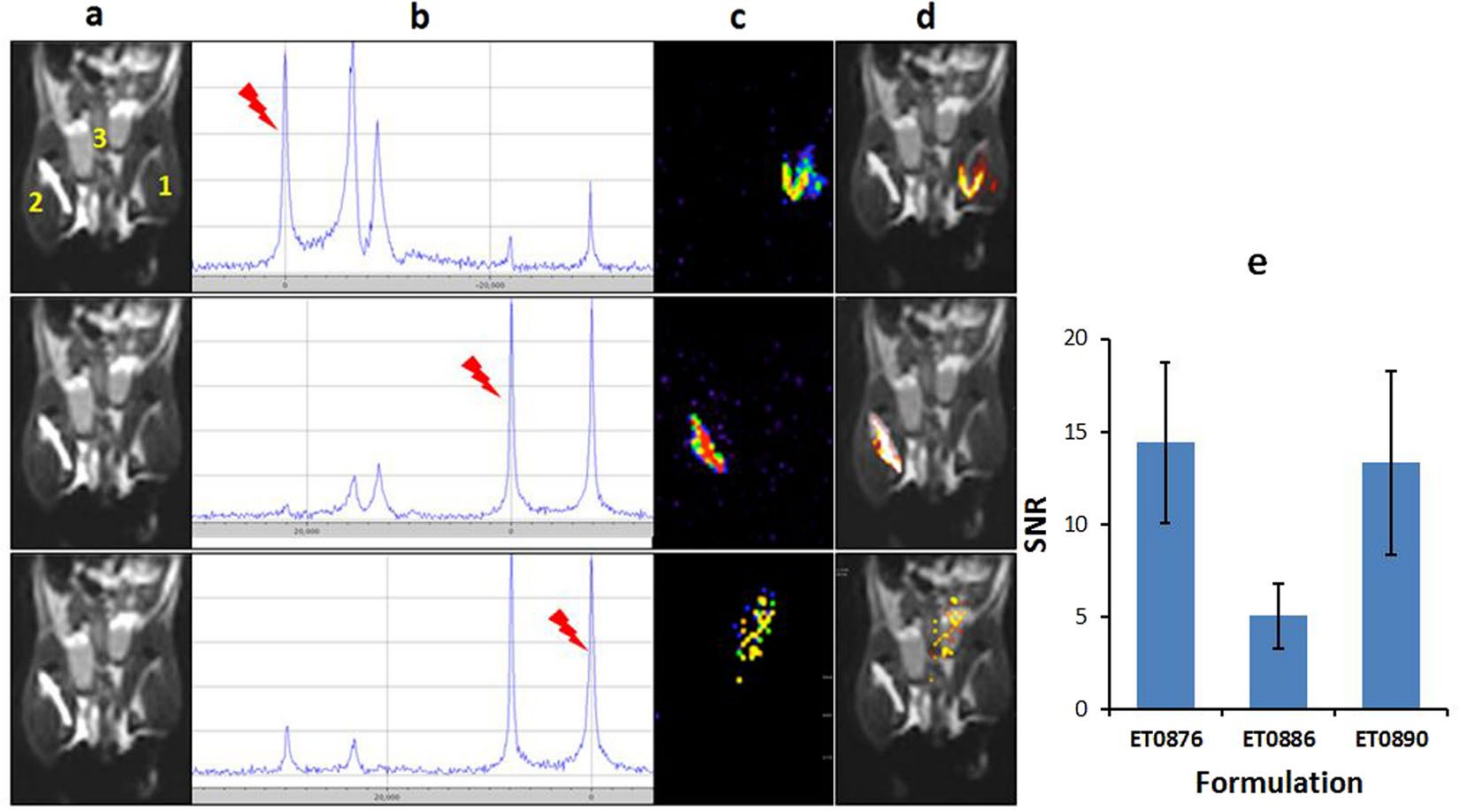
Evaluation of the spectral ^19^F MR imaging potential probes in an *in vivo* environment. (**a**) TurboRARE T_2_^1^H MR (same scan parameters as in Fig. 4 above) anatomy image of a isoflrane anesthetized mouse injected intramuscularly with formulations **ET0876** (right thigh, position 2), and **ET0890** (left thigh, position 1), and subcutaneously **ET0886** (abdomen, position 3). (**b**) Single pulse ^19^F MR scan showing resonance frequencies of ^19^F species within the subject in the scanner with frequencies of interest highlighted by the red pointer. (**c**) ^19^F MR image obtained from a ^19^F MSME scan protocol (same scan parameters as in Fig. 4 above), of the selected frequency. (**d**) overlay of ^19^F MR image over anatomy image reports on location of each probe. (**e**) SNR of formulations *in vivo* (n = 4).

Preliminary evaluation of imaging using these agents in an *in vivo* environment was performed. 50 µL of each formulation were injected as follows: intramuscularly (**ET0876** in right thigh and **ET0890** in the left thigh), and subcutaneously (**ET0886** in the abdominal area), in C57BL6 mice (n = 4). Each animal was anesthetized with isoflurane, placed in the magnet, and imaged using the same scan protocol as the phantoms. First, a TurboRARE T_2_^1^H scan, with the lower torso of the animal in the field of view to show the general anatomy (the numbers 1, 2 and 3 indicate the locations in which the probes were administered, Fig. 5a), was performed. When the animal was imaged using the ^19^F MSME scan sequence with the base frequency set to the resonance frequency of highlighted peak on the Single pulse ^19^F spectrum (Fig. 5b), only a single ^19^F signal, corresponding to that peak was generated on the ^19^F MR image (Fig. 5c). Overlay of the ^19^F image over the anatomy image showed the exact location of each ^19^F spot (Fig. 5d). The average SNR of the porobes within the muscle was determined to be about 15 and about 5 for the probe in the subcutaneous space (Fig. 5e). The more diffuse signal and low SNR in the abdominal area was attributed to faster diffusion of the nanoparticles within the subcutaneous space compared to the muscle.

## Discussion

^19^F MRI probes have the potential to simultaneously profile multiple molecular species/disease activity and disease sub-types within a target volume. Currently used PFCs and PFPEs lack flexibility in formulation and images often bear chemical shift artifacts due to the presence of non-identical fluorine nuclei on the probe molecules. Several strategies aimed at addressing some of these limitations are under investigation and well documented in a recent review^5^. Achieving a safely injectable formulation with any of these strategies remains a tall order. Partlow *et al*. have previously reported the ability to simultaneously track multiple targets *in vivo* using unique ^19^F MR signatures of PFC nanobeacons^14^, the formulation of which have similar limitations as the PFCs and PFPEs.

There is no previous report of hydrophilic fluorinated molecules designed with the goal of solving the formulation problem associated with current ^19^F MRI probes. Previous attempts at liposome formulations have focused on the use of perfluorofatty acids or sulfonate amphiphiles to incorporate flourine in the bilayer of the particle^15,16^, or the use of inorganic fluoride^17^. These approaches suffer from limitted payload capacity. For instance, in a liposome formulation with a mean particle diameter of 150 nm, only a fraction of the ~350,000 total possible molecules in the bilayer (including phospholipids and cholesterol) can be the fluorinated amphiphile. Similarly, the number of water soluble ionic fluorides that can be encapsulated in a liposome is limited by the osmolality of the solution, and potential toxicity of the salt. However, a formulation with a similar particle size distribution and an aqueous interior loaded with nonionic hydrophilic molecules with osmolality of 300 mOsmol/kg can carry ~10^6^ hydrophilic molecules per particle.

Our hydrophilic organofluorine molecules approach employs facile synthetic routes to water-soluble molecules with magnetically equivalent fluorine atoms which generate ^19^F MRI images with no chemical shift artifacts. When combined with the liposome nanoparticle platform, it can access the broad chemical shift spectrum of organofluorine species, enabling numerous probes with unique ^19^F MR signatures. Such probes have the potential to enable noninvasive simultaneous visualization of multiple hot spots within the same region of interest by ^19^F MRI as demostrated in both our phantom and preliminary *in vivo* assessments of the three formulations.

Isoflurane, the commonly used inhalable anesthesia in small animal studies, is fluorinated, highly lipophilic, and readily absorbed by adipose tissue *in vivo*. The ^19^F NMR chemical shifts of its two sets of fluorine nuclei are in the same vicinity as those of most PFCs and PFPEs. These can interfere with any signal from a PFC or PFPE probe, complicating data interpretation. While this problem can be addressed by using non-fluorinated injectable anesthetics such as ketamine and xylazine, these have other problems associated with them including effects on hemodynamics in key organs^18^ and a more complicated work flow. ^19^F MRI probes that are not affected by isoflurane are highly desirable. This new approach allows for facile access to such probes.

The Rose criterion suggests a SNR of ~4 is needed to be able to distinguish image features at 100% certainty^19^. The **ET0876** formulation with a ^19^F content of 22.7 mg ^19^F atoms/mL, borne on a molecule with a molecular weight of 472.4 g/mol and generates a phantom image with an SNR of 38. Dimers and oligomers of this molecule as well as the others are synthetically accessible, suggesting that an increase in the ^19^F content of each formulation is feasible (though the number of monomers units per oligomer may be limited by viscosity of the corresponding aqueous solution with increasing molecular weight). Moreover, the dilution studies show that a SNR of >4 is obtained from a 10 minutes scan with as few as 2.5 × 10^13^ particles/mL of this formulation. This concentration represents an 8-fold dilution of the stock solution or an injection volume of about 250 µL in a 25 g mouse (blood volume ~2 ml), suggesting that these probes may be amenable vascular imaging. The consistently high T_2_ values (all >100 ms) allow for several echoes per excitation. This explains the exquisite signals obtained with the MSME scan protocol for all three formulations. They also suggest the possibility of applying both 2D and 3D TurboRARE scan protocols to obtain images with high SNR using these formulations and are subject to subsequent investigations.

In summary, readily available hydrophilic molecules and small organofluorine moieties were condensed to generate nonionic hydrophilic fluorinated molecules with unique ^19^F MR signatures. These were used to fabricate stable liposome formulations, and preliminary evaluation in both *in vitro* and *in vivo* environments demonstrate that spectral ^19^F MRI can be used to report on the location of each probe within the same region of interest without interference from the others or Isoflurane. These results, in addition to the growing interest in ^19^F MRI molecular imaging suggest that this is an excellent approach to harness the broad chemical shift spectrum of ^19^F molecular species to generate a myriad of unique probes for ^19^F MRI. Future efforts will be focused on targeted formulations which can home-in, and report on specific *in vivo* targets upon intravenous administration. In addition, increased sensitivity on a per particle basis will be sought by utilizing dimers and oligomers of these base molecules.

## Materials and Methods

Chemical synthesis and characterization of intermediates and final compounds are described in supplementary information S1.

### Preparation of liposomes

A lipid mixture consisting of 1,2-dipalmitoyl-sn-glycero-3-phosphocholine (DPPC), cholesterol, and 1,2-distearoyl-sn-glycero-3-phosphoethanolamine-N-[methoxy (polyethylene glycol)-2000] (DSPE-mPEG-2000) in a molar ratio of 55∶40∶5 was used to make liposomes. The lipid mixture, weighed to result in a final lipid concentration of 150 mM was dissolved in 100% ethanol, at a volume of 10% of desired final volume. The mixture was then warmed in a water bath, maintained at 60–64 °C, to obtain a clear solution. After the lipids had completely dissolved, a pre warmed solution of the desired compound dissolved in histidine/saline buffer (10 mM with no pH adjustment) was added: for **ET0863** and **ET0876**, the concentration of the compound was 1.0 M while for **ET0886** and **ET0890** the concentrations were 0.8 and 0.5 M respectively. The resulting mixture was incubated at 60–64 °C for 45 min and the spontaneously formed multilamellar liposomes extruded on a Lipex thermoline extruder (Northern Lipids Inc., Canada), beginning with five passes through a 400 nm Nuclepore membrane (Waterman, Newton, MA) followed by eight passes through a 100 nm membrane. The resulting preparation was subjected to diafiltration through a 500 kD membrane (Spectrum Labs, Rancho Dominguez, CA) for 10 volume exchanges to practically eliminate any unencapsulated compound. Mean particle size was determined by Dynamic Light Scattering on a BI-90 goniometer/autocorrelator system at 90° using a 532 nm solid state laser source (Brookhaven Instruments Corp, Holtville, NY), and the final ^19^F content determined by comparing ^19^F NMR integrals against a standard solution prepared from analytical grade trifluoroacetic acid (Sigma Aldrich).

### Leak Test in Bovine Plasma

The percentage leak in bovine plasma (Sigma-Aldrich, St. Louis, MO) was determined by diluting 70 µL of the liposomal prep in 500 µL of bovine plasma. The solution was then incubated for 90 ± 5 minutes at 37 ± 1 °C. After the incubation was completed, 400 µL of the sample was diluted in 2.4 mL of 0.9% saline. 2 mL of the sample was then transferred into a 10,000 MWCO Vivaspin-2 centricon tube (Sartorius, Bohemia, New York) and centrifuged at 1100 rpm for 15 minutes. The filtrate was then analyzed for fluorine by NMR integration against a standardized trifluoroacetic acid solution. The % leak was determined by comparing the concentration of fluorine that leaked out to the total concentration of fluorine in the formulation.

### MRI acquisition and data processing

All MRI scans were performed on a 9.4 T Bruker small animal MR scanner equipped with a ^1^H/^19^F dual-tunable volume RF coil (35 mm inner diameter, 50 mm length; Rapid Biomed, Würzburg, Germany), located in the Small Animal Imaging Facility (SAIF) at Texas Children’s Hospital. ^19^F images of both phantoms and mice were acquired with an MMSE scan protocol (Excitation bandwidth = 2000 Hz, TR = 2000 ms, TE = 8.95 ms, scan time = 10 min 40 s). ^1^H images were acquired with a TurboRARE T_2_ scan protocol (TR = 2500 ms, TE = 11 ms, RARE factor = 4, scan time = 5 min 20 s). Mice were anesthetized by exposure to Isoflurane prior to injection of probes and maintained under anesthesia for the duration of the experiment, as well as a temperature of 37 °C using a temperature controlled air-flow system. Dicoms obtained from scans were processed using the OsiriX v.5.8.5 software (Pixmeo SARL, Bernex, Switzerland).

### Relaxation times

Relaxation times T_1_ and T_2_ were estimate using similar scan sequences and parameters as reported by Tirotta *et al*.^12^: For T_1_ relaxation times, a saturation recovery (RAREVTR) sequence with the following parameters (FOV = 5*5 cm^2^; Matrix = 32*32; Slices = 14; ST = 0.7 mm; TE = 11 ms; TR = 10000, 5000, 2500, 1500, 800, 400, 200, 100 ms; Rare Factor = 2; BW = 15 kHz; NA = 50; Dummy Scans (DS) = 0). For T2 relaxation times, a Multi Slice Multi Echo (MSME) sequence with the following parameters (FOV = 5*5 cm^2^; Matrix = 32*32; Slices = 1; Slice Thickness = 10 mm; TE = 11 ms; TR = 5000 ms; Number of echos = 40; BW = 15 kHz; NA = 100; DS = 0). Image sequence analysis in ParaVision 5.1 software was used to convert the raw data to numerical values.

### Ethical approval

All applicable international, national, and/or institutional guidelines for the care and use of animals were followed. Animal handling procedures were carried out following approved protocols by the Baylor College of Medicine Institutional Animal Care and Use Committee (IACUC).

## Electronic supplementary material

Supplementary Information-S1
