# Supplementary material for: Hydrophilic fluorinated molecules for spectral 19F MRI
Source: Sci Rep. 2018 Feb 13;8:2889. doi: 10.1038/s41598-018-21178-3 (PMC5811496; doi:10.1038/s41598-018-21178-3)
Supplement: Supplementary file 1 — Supplementary Information-S1 [file 41598_2018_21178_MOESM1_ESM.pdf]

Hydrophilic fluorinated molecules for spectral ^{19}F MRI

Eric A. Tanifum,*,[a],[b] Chandreshkumar Patel,[a][c] Matthew E. Liaw,[d] Robia G. Pautler,[b],[e] Ananth V. Annapragada[a],[b]

[a] Department of Pediatric Radiology, Texas Children's Hospital, Houston, TX 77030, USA

[b] Department of Radiology, Baylor College of Medicine, Houston, TX 77030, USA

[c] Current address: Department of Biomedical Engineering, UT Southwestern Medical Center, Dallas, TX 75390, USA.

[d] School of Medicine, Baylor College of Medicine, Houston, TX 77030, USA

[e] Department of Molecular Physiology and Biophysics, Baylor College of Medicine, Houston, TX 77030, USA.

[*] Corresponding author: Department of Pediatric Radiology, Texas Children's Hospital, 1102 Bates St, Ste 850, Houston, TX 77030, USA. E-mail: eatanifu@texaschildrens.org. Tel: (832)-824-0866 Fax: (832)-825-0260

Supplementary Information – S1

Chemical synthesis and characterization of intermediates and final compounds.

General Procedures. 2,2,3,3-Tetrafluoro-1,4-butanediol was purchased from Exfluor Research Corp., Round Rock, TX, USA, and 1,4-bis(bromomethyl)-3,4,5,6-tetrafluorobenzene (**7**) was purchased from Molport, Riga, Latvia. All other reagents, including redistilled analytical grade trifluoroacetic acid, were obtained from Sigma-Aldrich and used without further purification. Proton nuclear magnetic resonances (^{1}H NMR) spectra were recorded at 600 MHz on a Bruker 600 NMR spectrometer or at 300 MHz on a Bruker 300 NMR spectrometer. Carbon nuclear magnetic resonances (^{13}C NMR) spectra were recorded at 150 MHz on a Bruker 600 NMR spectrometer or at 75 MHz on a Bruker 300 NMR spectrometer. Fluorine nuclear magnetic

resonances (^{19}F NMR) spectra were recorded at 282 MHz on a Bruker 300 NMR spectrometer. Chemical shifts are reported in parts per million (ppm) from an internal standard acetone (2.05 ppm), chloroform (7.26 ppm), or water (4.79 ppm) for ^{1}H NMR; and from an internal standard of either residual acetone (206.26 ppm), chloroform (77.00 ppm), or dimethyl sulfoxide (39.52 ppm) for ^{13}C NMR. NMR peak multiplicities are denoted as follows: s (singlet), d (doublet), t (triplet), q (quartet), p (pentet), bs (broad singlet), dd (doublet of doublet), tt (triplet of triplet), ddd (doublet of doublet of doublet), and m (multiplet). Coupling constants (*J*) are given in hertz (Hz). High resolution mass (HRMS) spectra were obtained from the Mass Spectrometry Unit of the Bioscience Research Collaborative at Rice University, Houston, Texas. Thin layer chromatography (TLC) was performed on silica gel 60 F254 plates from EMD Chemical Inc. and components were visualized by ultraviolet light (254 nm) and/or phosphomolybdic acid, 20 wt% solution in ethanol. SiliFlash silica gel (230-400 mesh) was used for all column chromatography.

Procedure for compound 1. To a solution of compound glycerol (50.00 g, 542.9 mmol), in acetone (2L), was added 2,2-dimethoxypropane (100 mL, 806.0 mmol), followed by *p*-toluenesulfonic acid (1.03 g, 5.429 mmol). The mixture was stirred at ambient temperature for 12 hours, followed by addition of solid anhydrous potassium carbonate (6.00 g, 4.34 mmol). The mixture was stirred for a further 30 mins followed by filtration of solids. The filtrate was concentrated by rotary evaporation to obtain the desired acetonide **1,** quantitatively as a clear residue, used in subsequent steps without further purification. ^{1}H NMR (300 MHz, $CDCl_3$) δ 4.4.14 (p, *J* = 4.8 Hz, 1H), 3.96 (dd, *J* = 8.4, 6.6 Hz, 1H), 3.69 (dd, *J* = 9.6, 6.0 Hz, 1H), 3.60 (dd, *J* = 12.0, 4.8 Hz, 1H), 3.52 (dd, *J* = 12.0, 4.8 Hz, 1H), 3.13 (s, 1H/OH), 1.35 (s, 3H), 1.29 (s, 3H); ^{13}C NMR ($CDCl_3$, 150 MHz) δ 109.30, 76.21, 65.81, 62.94, 26.59, 25.18.

Procedure for compound 2: To a solution of compound **1** (50.00 g, 378.3 mmol), in pyridine (500 mL), cooled in an ice/water bath, was added *p*-toluenesulfonyl chloride (93.70 g, 491.8

mmol). The mixture was stirred at 4 °C for 12 hours, followed by removal of pyridine by rotary evaporation. The residue was diluted with 500 mL 1N HCl and extracted (3X) with diethyl ether. The combined organic extracts were rinsed with brine, dried over $MgSO_4$ and concentrated by rotary evaporation to obtain a clear residue which was dissolved in DMF (400 mL) followed by addition of NaN_3 (92.00 g, 1.4 mol). The ensuing mixture was heated at 70 °C for 12 hours, after which it was cooled down to ambient temperature and filtered through a pad of celite. The filtrate was diluted with water and extracted (3X) with diethyl ether. The combined organic extracts were rinsed with brine, concentrated by evaporation to obtain a clear residue. This was subjected to column chromatography on silica gel eluted with a diethyl ether/pentane mixture (1:9), to obtain **2** as clear oil (32.80 g, 65 % yield over two steps). ^{1}H NMR (300 MHz, $CDCl_3$) δ 4.21 (p, *J* = 6 Hz, 1H), 3.99 (dd, *J* = 8.4, 6.0 Hz, 1H), 3.70 (dd, *J* = 8.4, 6.0 Hz, 1H), 3.33 (dd, *J* = 13.2, 4.8 Hz, 1H), 3.23 (dd, *J* = 13.2, 4.8 Hz, 1H), 1.40 (s, 3H), 1.30 (s, 3H); ^{13}C NMR (75 MHz, $CDCl_3$) δ 109.81, 74.53, 66.47, 52.73, 26.50, 25.12.

Procedure for compound 3: To a suspension of NaH (60% in mineral oil, 3.458 g, 144.1 mmol), in THF (500 mL), cooled in an ice/water bath was added diacetone glucose (25.00 g, 96.05 mmol). The mixture was stirred at 0 °C for 30 mins, followed by addition of propargyl bromide (17.1 mL, 115.3 mmol), and the ensuing mixture allowed to warm to ambient temperature overnight. Unreacted NaH was quenched by pouring the reaction mixture into crushed ice and further diluted with water, resulting in two phases. The organic phase was removed and the aqueous phase extracted (2X) with diethyl ether. The combined organic extracts were rinsed with brine, dried over $MgSO_4$ and concentrated. The ensuing brown residue was subjected to column chromatography on silica gel eluted with 10% ethyl acetate/hexanes mixture to obtain compound **3** as pale yellow oil (16.50 g, 55% yield). ^{1}H NMR (600 MHz, $CDCl_3$) δ 5.87 (d, *J* = 3.6 Hz, 1H), 4.62 (d, *J* = 4.2 Hz, 1H), 4.28 (m, 3H), 4.13 (dd, *J* = 7.2, 3.0 Hz, 1H), 4.08 (m, 2H), 3.98 (dd, *J* = 8.4, 5.4 Hz, 1H), 2.49 (t, *J* = 2.4 Hz, 1H), 1.49 (s, 3H), 1.41 (s, 3H),

1.34 (s, 3H), 1.31 (s, 3H); ^{13}C NMR (150 MHz, $CDCl_3$) δ 111.85, 108.99, 105.20, 82.82, 81.52, 80.99, 79.26, 74.96, 72.51, 67.17, 58.08, 26.81 (2C), 26.23, 25.36.

Procedure for compound 4. To a stirred mixture of propargylamine (4.0 mL, 62.45 mmol) and diisopropylethylamine (21.8 mL, 124.9 mmol), in THF (150 mL) at 0 °C was added trifluoroacetic anhydride (13.1 mL, 93.68 mmol) drop wise. The mixture was allowed to warm to room temperature over 3 hours, at which point the reaction was judged complete by TLC. The mixture was poured into 1N HCl solution (300 mL) an extracted (3X) with ethyl acetate. The combined organic extracts were rinsed sequentially with saturated $NaHCO_3$ solution and brine, dried over Na_2SO_4 and concentrated to obtain the crude product. This was further purified by column chromatography on silica gel eluted with 15-30% ethyl acetate/hexanes gradient to afford **4** as pale yellow oil (8.680 g, 92% yield). ^{1}H NMR (300 MHz, $CDCl_3$) δ 7.34 (bs, NH), 4.14 (s, 2H), 2.32 (s, 1H); ^{19}F NMR (282 MHz, $CDCl_3$) δ -76.11; ^{13}C NMR (75 MHz, $CDCl_3$) δ 157.34 (q, J = 9.4 Hz), 115.59 (q, J = 71.3 Hz), 72.84, 29.59.

Procedure for compound 6. Compound **5** (10.00 g, 61.70 mmol) was added to a suspension of NaH (60% in mineral oil, 3.702 g, 154.2 mmol), in THF (300 mL), cooled in an ice/water bath. The mixture was stirred at 0 °C for 30 mins, followed by addition of propargyl bromide (22.1 mL, 148.1 mmol), and the ensuing mixture allowed to warm to ambient temperature overnight. The reaction was quenched by pouring into crushed ice, extracted with ether, rinsed with brine, and dried over anhydrous Na_2SO_4. Following filtration, the solvent was removed *in vacuo* to give a crude mixture which was purified by column chromatography on silica gel eluted with 10% ethyl acetate/hexanes to obtain **6** (13.80 g, 94% yield) as a colorless oil. ^{1}H NMR (300 MHz, $CDCl_3$) δ 4.29 (d, J = 2.4, 4H), 3.99 (tt, J = 14.2, 2.6 Hz, 4H), 2.54 (t, J = 2.4, 2H); ^{19}F NMR (282 MHz, $CDCl_3$) δ -121.69; ^{13}C NMR (75 MHz, $CDCl_3$) δ 115.96 (tt, J = 62.8, 7.7 Hz), 78.00, 75.97, 65.94 (t, J = 6.4 Hz), 59.24.

Procedure for compound 8. NaN_3 (19.35 g, 297.9 mmol) was added to a solution of **7** (10.00 g, 29.77 mmol) in anhydrous DMF (150.0 mL) and the resulting mixture heated at 70 °C for 5 h, after which it was cooled to room temperature. The solids were filtered off and the filtrate concentrated *in vacuo.* The ensuing residue was diluted with water and extracted (3X) with diethyl ether. The combined organic extracts were dried over Na_2SO_4 and concentrated to obtain **8** as a pale orange solid (7.580 g, 98% yield), which gave a single spot on TLC, and was used in the next step without further purification. ^{1}H NMR (300 MHz, $CDCl_3$) δ 4.51 (s, 4H); ^{19}F NMR (282 MHz, $CDCl_3$) δ -142.24; ^{13}C NMR (75 MHz, $CDCl_3$) δ 144.94 (dm, *J* = 50.5 Hz, 4C), 115.13 (m, 2C), 41.90 (t, *J* = 2.3 Hz).

Procedure for compound 9. To a stirred mixture of glycerol (31.54 g, 342.5 mmol), *p*-anisaldehyde (50.0 mL, 410.9 mmol), 4 Å molecular sieves (20.0 g) in anhydrous DMF (200 mL) was added p-toluene sulfonic acid (3.250 g, 17.09 mmol). The mixture was stirred for 12 h, after which it was poured into saturated $NaHCO_3$ solution and extracted with diethyl ether. The combined organic extracts were rinsed with brine, and dried over anhydrous Na_2SO_4. Following filtration, the solvent was removed *in vacuo* to give a crude mixture which was purified by chromatography on silica gel eluted with 30-50% ethyl acetate/hexanes gradient. Fractions containing **9** gave a white solid upon concentration (36.5 g, 51% yield). ^{1}H NMR (300 MHz, $CDCl_3$) δ 7.43 (d, *J* = 8.7 Hz, 2H), 6.93 (d, *J* = 8.7 Hz, 2H), 5.34 (s, H), 4.20 (dd, *J* = 10.5, 4.8 Hz, 2H), 3.85 (m, 1H), 3.78 (s, 3H), 3.48 (t, *J* = 11.1, 2H), 3.36 (m, 1H); ^{13}C NMR (75 MHz, $CDCl_3$) δ 160.17, 129.92, 127.57, 113.78, 100.99, 71.64, 60.98, 55.34.

Procedure for compound 10. Alcohol **9** (8.4 g, 61.7 mmol) was added to a suspension of NaH (60% in mineral oil, 1.4 g, 58.0 mmol), in THF (250 mL), cooled in an ice/water bath. The mixture was stirred at 0 °C for 30 mins, followed by addition of 3,5-bis(trifluoromethyl)benzyl bromide (10 g, 32.6 mmol), and the ensuing mixture allowed to warm to ambient temperature for 12 h. The reaction was quenched by pouring into crushed ice, extracted with ether, rinsed with

brine, and dried over anhydrous Na_2SO_4. Following filtration, the solvent was removed *in vacuo* to give a crude mixture. This was purified by chromatography on silica gel with 5-10% ethyl acetate/hexanes gradient as eluent, to obtain **10** (13.4 g, 96% yield) as a white solid. ^{1}H NMR (300 MHz, $CDCl_3$) δ 7.88 (s, 1H), 7.82 (s, 2H), 7.46 (d, *J* = 8.6, 2H), 6.95 (d, *J* = 8.6, 2H), 5.44 (s, 1H), 4.70 (s, 2H), 4.45 (dd, *J* = 8.4, 4.5 Hz, 2H), 3.88 (m, 1H), 3.83 (s, 3H), 3.70 (m, 2H); ^{19}F NMR (282 MHz, $CDCl_3$) δ -62.90; ^{13}C NMR (75 MHz, $CDCl_3$) δ 160.18, 140.74, 131.80 (q, *J* = 33.8 Hz, 2C), 130.02, 127.43, 127.17 (d, *J* = 3 Hz), 123.32 (q, *J* = 270.8 Hz), 121.71 (p, *J* = 3.8 Hz), 69.98, 69.82, 68.86, 55.21.

Procedure for compound 11. Compound **10** (12.5 g, 28.6 mmol) was dissolved in 80% AcOH/H_2O mixture (200 mL), by warming in a water bath at 50 °C. The solution was then allowed to stir at room temperature for 12 h. The solvents were removed by rotary evaporation and the resulting residue chromatographed on silica gel eluted with 40-60% ethyl acetate/hexanes gradient to diol **11**, as clear oil (8.3 g, 91% yield). ^{1}H NMR (300 MHz, $CDCl_3$) δ 7.83 (s, 2H), 7.81 (s, 1H), 4.76 (s, 2H), 3.85 (dd, *J* = 11.7, 4.8 Hz, 2H), 3.76 (dd, *J* = 11.7, 4.8 Hz, 2H), 3.60 (p, *J* = 4.5, 1H), 3.50 (bs, 2H); ^{19}F NMR (282 MHz, $CDCl_3$) δ -62.98; ^{13}C NMR (75 MHz, $CDCl_3$) δ 140.81, 132.10, 131.67 (q, *J* = 8.3 Hz, 2C), 127.38 (d, *J* = 3.0 Hz) , 123.26 (q, *J* = 271.5 Hz), 121.56 (p, *J* = 3.0 Hz), 114.34, 80.02, 70.31, 61.96.

Procedure for compound 12. To a solution of compound **11** (8.0 g, 25.6 mmol), in pyridine (150 mL), cooled in an ice/water bath was added *p*-toluenesulfonyl chloride (14.7 g, 76.9 mmol). The mixture was stirred at 4 °C for 12 hours, followed by removal of pyridine by rotary evaporation. The residue was diluted with 200 mL dichloromethane, rinsed with 1N HCl solution, dried over $MgSO_4$ and concentrated by rotary evaporation to obtain a clear residue (16.1 g). This was dissolved in DMF (150 mL) followed by addition of NaN_3 (16.6 g, 256.2 mol). The ensuing mixture was heated at 70 °C for 5 hours, after which it was cooled down to

ambient temperature and filtered through a pad of celite. The filtrate was diluted with water and extracted (3X) with diethyl ether. The combined organic extracts were rinsed with brine, concentrated by rotary evaporation to obtain a clear residue. This was subjected to column chromatography on silica gel eluted with 10% ethyl acetate/hexanes mixture (1:9), to obtain **12** as clear oil (7.9 g, 84 % yield over two steps). ^{1}H NMR ($CDCl_3$, 300 MHz) δ 7.88 (s, 2H), 7.86 (s, 1H), 4.84 (s, 2H), 3.78 (p, *J* = 5.1, Hz, 1H), 3.49 (d, *J* = 5.1, 4H)); ^{19}F NMR (282 MHz, $CDCl_3$) δ -63.03; ^{13}C NMR (75 MHz, $CDCl_3$) δ 140.08, 131.78 (q, *J* = 33.0 Hz, 2C), 127.35, 123.29 (q, *J* = 271 Hz), 78.92, 70.94, 51.98.

Procedure for compound 13. To a solution of **4** (8.0 g, 52.9 mmol), **2** (10.0 g, 63.5 mmol), and sodium ascorbate (1.0 g, 5.3 mmol) in a mixture of methanol/ethyl acetate/water (5:2:2, 180 mL) was added $Cu(OAc)_2$ (528.6 mg, 2.6 mmol). The mixture was stirred at room temperature for 12 h then poured into a brine/water mixture (1:1, 200 mL), extracted with ethyl acetate (200 mL, 3 times). The combined organic phases were dried over Na_2SO_4, filtered, and upon concentration gave a white solid which was washed with a mixture of ethyl acetate/hexanes to obtain **13**, as a white crystalline solid (13.7 g, 84% yield). ^{1}H NMR (300 MHz, $CDCl_3$) δ 8.45 (s, NH), 7.80 (s, 1H), 4.61-4.39 (m, 5H), 4.13 (m, 1H), 3.75 (m, 1H); ^{19}F NMR (282 MHz, $CDCl_3$) δ -75.82; ^{13}C NMR (75 MHz, $CDCl_3$) δ 157.52 (q, *J* = 35.25 Hz), 142.79, 124.43, 115.81 (q, *J* = 268.5 Hz), 110.34, 73.84, 66.26, 52.49, 34.81, 26.56, 25.08.

Procedure for compound 14. To a solution of **6** (6.0 g, 25.2 mmol), **2** (9.9 g, 63.0 mmol), and sodium ascorbate (1.0 g, 5.0 mmol) in a mixture of methanol/ethyl acetate/water (5:2:2, 180 mL), was added $Cu(OAc)_2$ (503 mg, 2.5 mmol). The mixture was stirred at room temperature for 12 h, then poured into a brine/water mixture (1:1, 200 mL), and extracted with ethyl acetate (200 mL, 3 times). The combined organic phases were dried over Na_2SO_4, filtered, and concentrated. The ensuing residue was chromatographed on silica gel eluted with 30-50% ethyl acetate/hexanes gradient, then a mixture of ethyl acetate/hexanes/methanol (5:4:1) to afford **14** as

a white solid (11.7 g, 98% yield). ^{1}H NMR (300 MHz, $CDCl_3$) δ 7.76 (s, 2H), 4.75 (s, 4H), 4.58-4.40 (m, 6H), 4.10 (dd, J = 10.5, 8.7 Hz, 2H), 3.95 (t, J = 14.1 Hz, 4H), 3.75 (dd, J = 8.7, 5.7 Hz, 2H); ^{19}F NMR (282 MHz, $CDCl_3$) δ -121.72; ^{13}C NMR (75 MHz, $CDCl_3$) δ 143.83, 124.34, 110.22, 73.97, 66.91 (t, J = 25.5 Hz), 66.40, 65.52, 52.36, 30.89, 26.64, 25.15.

Procedure for compound 15. To a solution of **8** (6.8 g, 26.1 mmol), **3** (23.4 g, 78.4 mmol), and sodium ascorbate (1.0 g, 5.2 mmol) in a mixture of methanol/ethyl acetate/water (3:1:1, 200 mL) was added $Cu(OAc)_2$ (521.9 mg, 2.6 mmol). The mixture was stirred at room temperature for 12 h after which the solvent was stripped *in vacuo*. The residue was diluted with water (200 mL), extracted with ethyl acetate (200 mL, 3 times). The combined organic phases were rinsed with brine, dried over Na_2SO_4, filtered, and concentrated. The ensuing residue was chromatographed on silica gel eluted with 30-50% ethyl acetate/hexanes gradient, then a mixture of ethyl acetate/hexanes/methanol (60:35:5) to afford **15** as a clear viscous paste which upon cooling and vacuum drying, foams into a white glassy solid (23.3 g, 94% yield). ^{1}H NMR (300 MHz, $CDCl_3$) δ 7.73 (s, 2H), 5.86 (d, J = 3.6 Hz, 2H), 5.65 (s, 4H), 4.79 (dd, J = 15.0, 12.6 Hz, 4H), 4.60 (d, J = 3.6 Hz, 2H), 4.29 (m, 2H), 4.07 (m, 8H), 1.48 (s, 6H), 1.41 (s, 6H), 1.35 (s, 6H), 1.30 (s, 6H). ^{19}F NMR (282 MHz, $CDCl_3$) δ -140.86; ^{13}C NMR (75 MHz, $CDCl_3$) δ 145.59, 122.74, 114.74, 111.89, 105.20, 82.56, 81.87, 81.06, 72.30, 67.43, 63.98, 41.08, 26.85, 26.77, 26.18, 25.39.

Procedure for compound 16. To a solution of **12** (6.0 g, 16.3 mmol), **3** (12.2 g, 40.8 mmol), and sodium ascorbate (711.3 mg, 3.6 mmol) in a mixture of methanol/ethyl acetate/water (3:1:1, 200 mL) was added $Cu(OAc)_2$ (325.8 mg, 1.6 mmol). The mixture was stirred at room temperature for 12 h after which the solvent was stripped *in vacuo*. The residue was diluted with water (200 mL), extracted with ethyl acetate (200 mL, 3 times). The combined organic phases were rinsed with brine, dried over Na_2SO_4, filtered, and concentrated. The ensuing residue was chromatographed on silica gel eluted with 30-50% ethyl acetate/hexanes gradient, then a mixture of ethyl acetate/hexanes/methanol (60:35:5) to afford **16** as a clear viscous paste which upon

cooling and vacuum drying, foams into a white glassy solid (15.3 g, 97% yield). ^{1}H NMR (300 MHz, $CDCl_3$) δ 7.82 (s, 1H), 7.73 (s, 2H), 7.59 (s, 2H), 5.86 (d, *J* = 3.9 Hz, 2H), 4.80 (s, 4H), 4.59 (s, 4H), 4.52 (m, 4H), 4.29 (m, 2H), 4.07 (m, 8H), 1.48 (s, 6H), 1.39 (s, 6H), 1.30 (s, 6H), 1.29 (s, 6H). ^{19}F NMR (282 MHz, $CDCl_3$) δ -62.98; ^{13}C NMR (75 MHz, $CDCl_3$) δ 145.23, 139.24, 131.95 (q, *J* = 33.0 Hz), 127.23, 124.87, 124.28, 124.25, 122.12, 121.25, 111.88, 109.05, 105.20, 82.73, 82.70, 81.99, 81.04, 72.37, 71.29, 67.36, 63.95, 26.82, 26.76, 26.16, 25.41.

Final step to ET0863. To a solution of **13** (5.0 g, 16.2 mmol) in methanol (160 mL) was added TsOH (617.0 mg, 3.2 mmol). The mixture was stirred at room temperature for 12 h after which the solvent was stripped *in vacuo*. The residue was diluted with mixture of brine and saturated $NaHCO_3$ (1:1, 200 mL), extracted with ethyl acetate (200 mL, 3 times). The combined organic phases were dried over Na_2SO_4, filtered, and concentrated down to 100 mL. Upon adding a few drops of hexanes, the product precipitated out of solution and was filtered to obtain **ET0863** as a white solid (3.8 g, 87% yield). ^{1}H NMR (300 MHz, D_2O) δ 7.99 (s, H), 4.1 (s, 2H), 4.58 (dd, *J* = 8.7, 2.1 Hz, 1H), 4.44(dd, *J* = 8.7, 4.8 Hz, 1H), 4.13 (m, 1H), 3.65 (dd, *J* = 7.2, 3.0 Hz, 1H), 3.57 (dd, *J* = 7.2, 3.6 Hz, 1H); ^{19}F NMR (282 MHz, D_2O) δ -75.90; ^{13}C NMR (75 MHz, D_2O) δ 158.85 (q, *J* = 22.5 Hz), 142.90, 125.15, 115.75 (q, *J* = 172.5 Hz), 70.20, 62.60, 52.70, 34.50; HRMS *clcd* for $C_8H_{11}F_3N_4O_3^+$ m/z $(M+Na)^+$ 291.0664, found 291.0666.

Final step to ET0876. A solution of **14** (26.3 g, 47.6 mmol) in AcOH/H_2O mixture (2:1, 300 mL) was stirred at room temperature for 12 h after which the acid/water mixture stripped at high vacuum. The residue was azeotroped 3 times with 100 mL portions of toluene and then methanol. The resulting residue was dried under high vacuum overnight to obtain **ET0876** as transparent glue which solidified into a white wax (22.4 g, 99% yield) upon storage at 2-8℃, and remains solid when left at room temperature.). ^{1}H NMR (300 MHz, CD_3OD) δ 8.05 (s, 2H), 4.75 (s, 4H), 4.59 (dd, *J* = 14.3, 3.9 Hz, 2H), 4.44(dd, *J* = 14.3, 8.1 Hz, 2H), 4.13 (m, 2H), 4.03

(t, *J* = 13.8 Hz, 4H), 3.64 (dd, *J* = 11.7, 4.8 Hz, 2H), 3.56 (dd, *J* = 11.7, 6.0 Hz, 2H); ^{19}F NMR (282 MHz, CD_3OD) δ -121.57; ^{13}C NMR (75 MHz, CD_3OD) δ 143.00, 126.02, 70.22, 64.31, 62.64, 52.66, 48.84; HRMS *clcd* for $C_{16}H_{24}F_4N_6O_6^+$ m/z $[M+H]^+$ 473.1753, found 473.1758.

Final step to ET0886. To a solution of **15** (11.1 g, 13.0 mmol) in AcOH/H_2O mixture (8:2, 150 mL) was added 300 µL HCl and the resulting mixture heated at 70℃ for 12 h. The acid/water mixture was stripped *in vacuo* and the resulting residue azeotroped 3 times with 50 mL portions of toluene. The ensuing residue was diluted with water (100 mL), titrated to pH 3.2 with NaOH and freeze dried to obtain a white solid (8.9 g, 98% yield). Analytical samples were further purified by passing through a short pad of silica gel eluted with 10-40% MeOH/CH_2Cl_2 gradient. ^{1}H NMR (300 MHz, CD_3OD) δ 8.10 (s, 2H), 5.81 (s, 4H), 5.12 (d, *J* = 3.6 Hz, 1H), 4.96 (s, 4H), 4.52 (d, *J* = 7.8 Hz, 2H), 3.82 (m, 3H), 3.67 (m, 3H), 3.45 (m, 3H), 3.30 (m, 3H); ^{19}F NMR (282 MHz, CD_3OD) δ -143.39; ^{13}C NMR (75 MHz, CD_3OD) δ 145.87, 145.77, 123.93, 96.73, 92.62, 85.27, 82.48, 76.40, 74.83, 72.35, 71.58, 69.99, 69.93, 65.31, 65.17, 61.33, 61.21, 40.92; HRMS *clcd* for $C_{26}H_{32}F_4N_6O_{12}^+$m/z $[M+H]^+$ 697.2098, found 697.2103.

Final step to ET0890. To a solution of **16** (5.6 g, 5.8 mmol) in AcOH/H_2O mixture (8:2, 100 mL) was added 150 µL HCl and the resulting mixture heated at 70℃ for 12 h. The acid/water mixture was stripped *in vacuo* and the resulting residue azeotroped 3 times with 50 mL portions of toluene. The ensuing residue was diluted with water (100 mL), titrated to pH 3.2 with NaOH and freeze dried to obtain a white solid (4.4 g, 95% yield). Analytical samples were further purified by passing through a short pad of silica gel eluted with 10-40% MeOH/CH_2Cl_2 gradient. ^{1}H NMR (300 MHz, CD_3OD) δ 8.05 (s, 2H), 7.83 (s, 1H), 7.71 (s, 2H), 5.13 (d, *J* = 3.3 Hz, 1H), 4.98-4.51 (m, 8H), 3.82 (m, 3H), 3.68 (m, 3H), 3.50-3.22 (m, 7H); ^{19}F NMR (282 MHz, CD_3OD) δ -62.65; ^{13}C NMR (75 MHz, CD_3OD) δ 140.69, 131.95 (q, *J* = 33.0 Hz), 127.52, 124.91, 124.28, 121.52, 121.12, 96.75, 92.62, 85.19, 82.32, 82.27, 76.82, 79.39, 74.84, 72.35, 71.55,

70.29, 70.07, 69.98, 65.19, 65.10, 61.38, 61.29, 50.70; HRMS *clcd* for $C_{30}H_{36}F_6N_6O_{13}^+$ m/z $[M+H]^+$ 805.2456, found 805.2467.

Supplementary Information – S2

^1H, ^{19}F, and ^{13}C NMR spectra of intermediates and final compounds

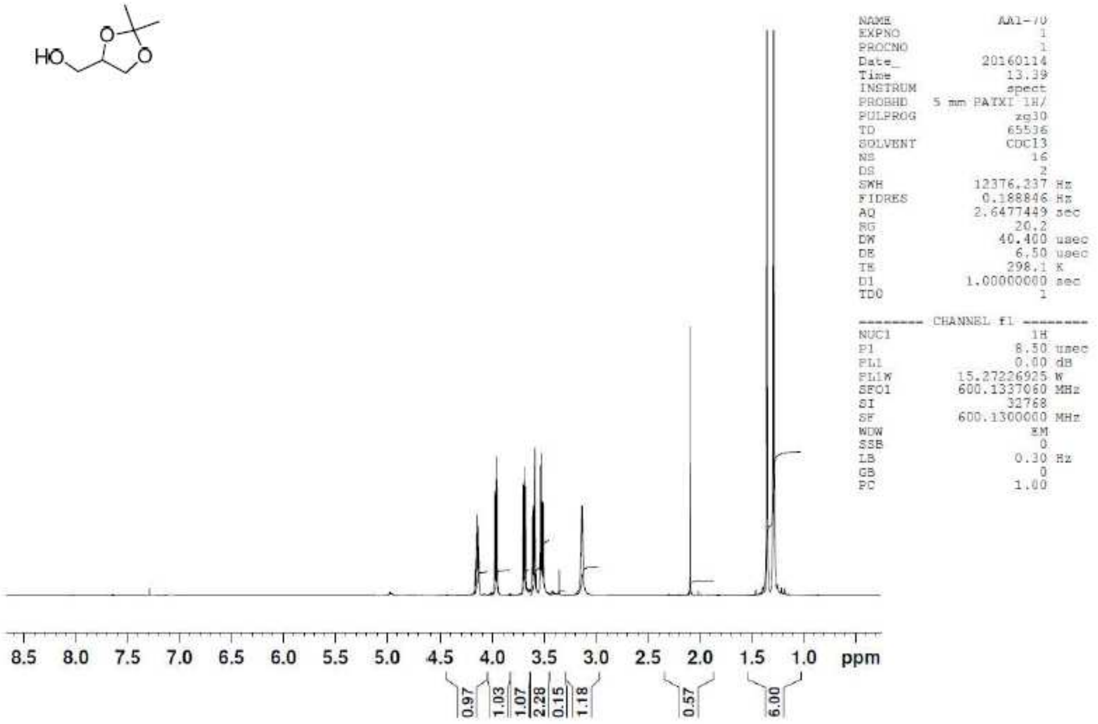

^1H NMR Spectrum of compound **1**

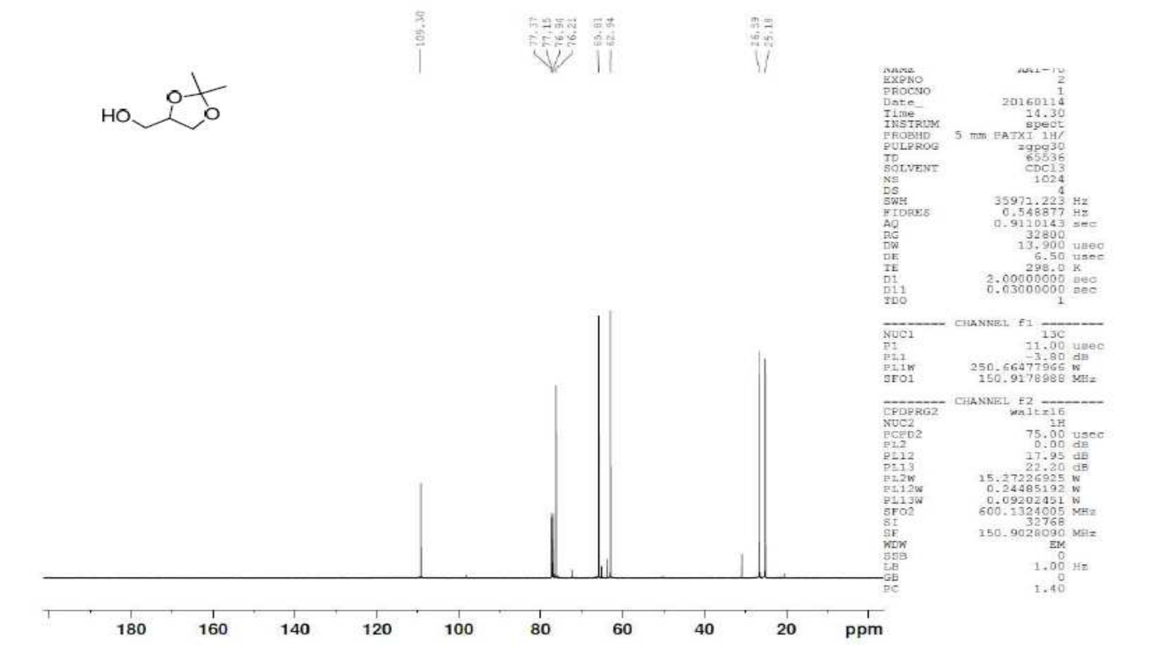

^{13}C NMR Spectrum of compound **1**

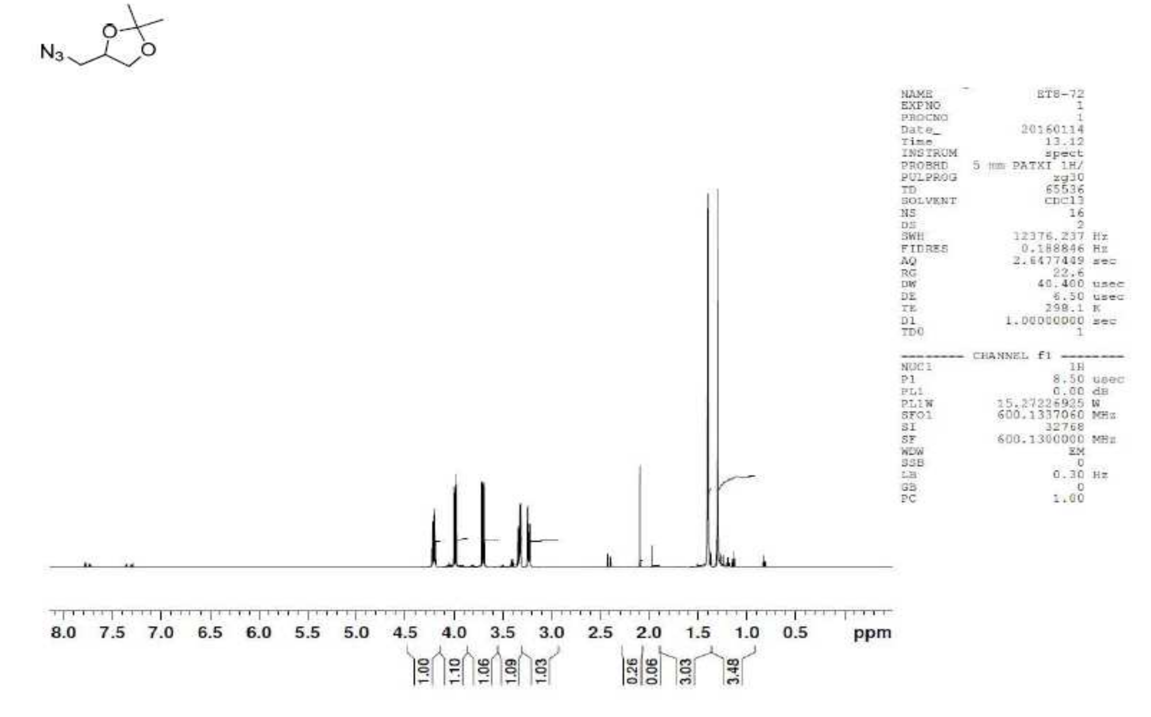

^{1}H NMR Spectrum of compound **2**

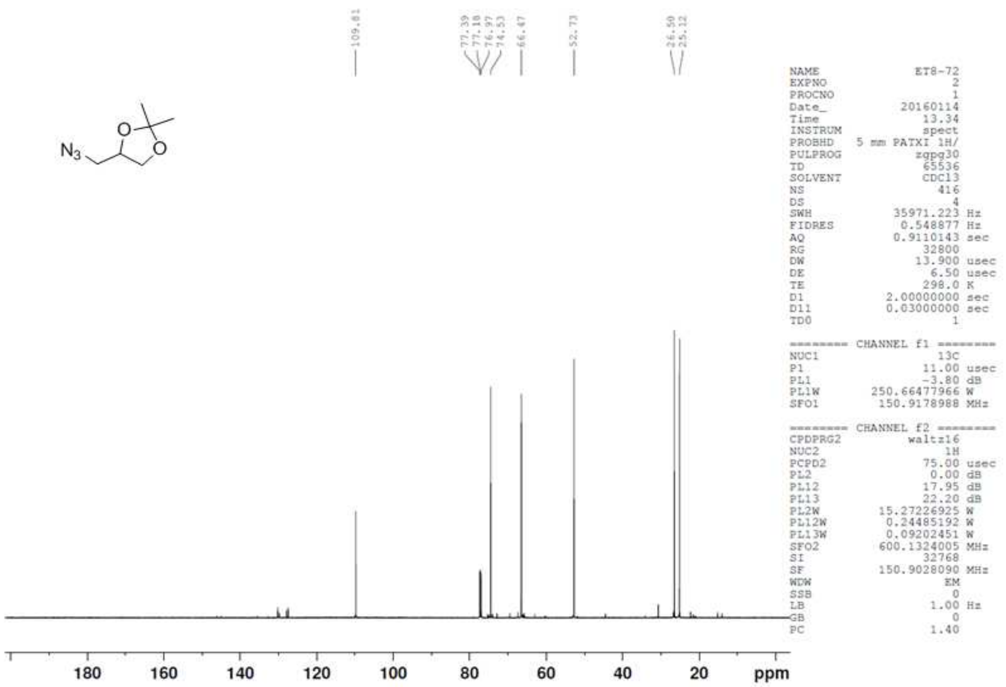

^{13}C NMR Spectrum of compound **2**

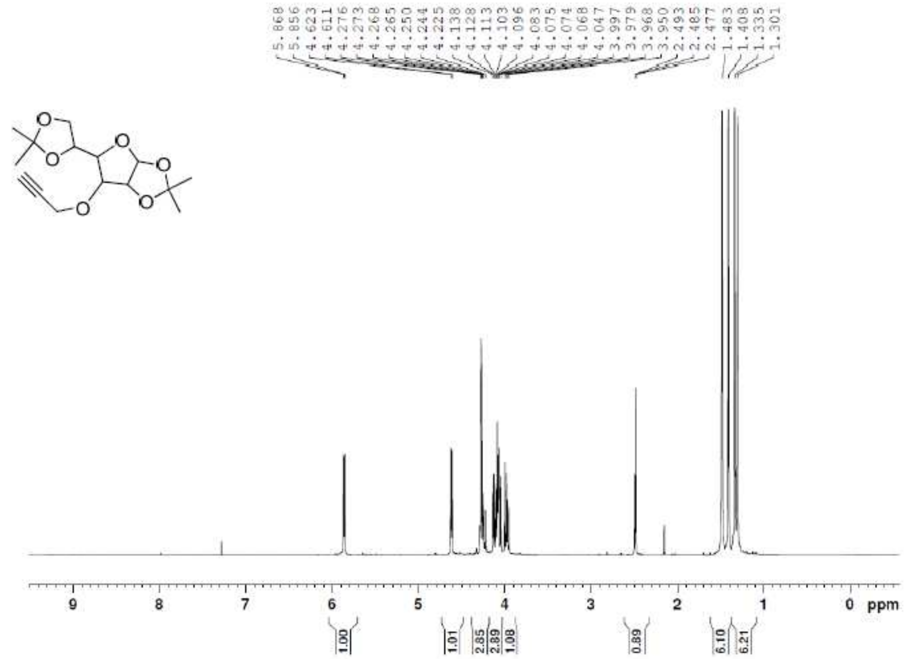

^{1}H NMR Spectrum of compound **3**

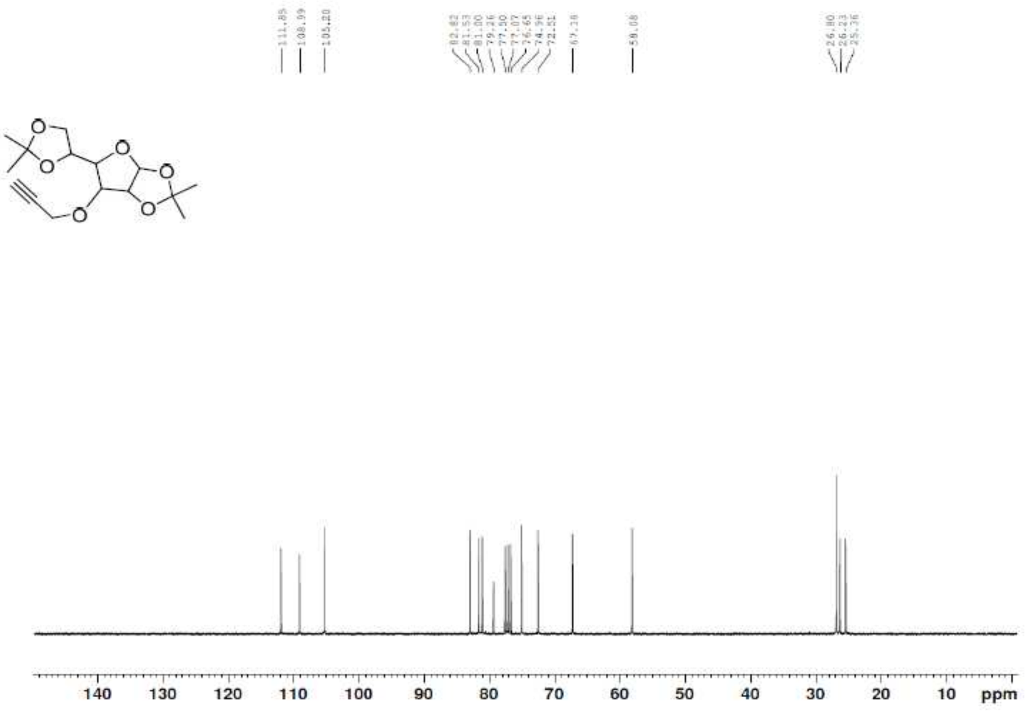

^{13}C NMR Spectrum of compound **3**

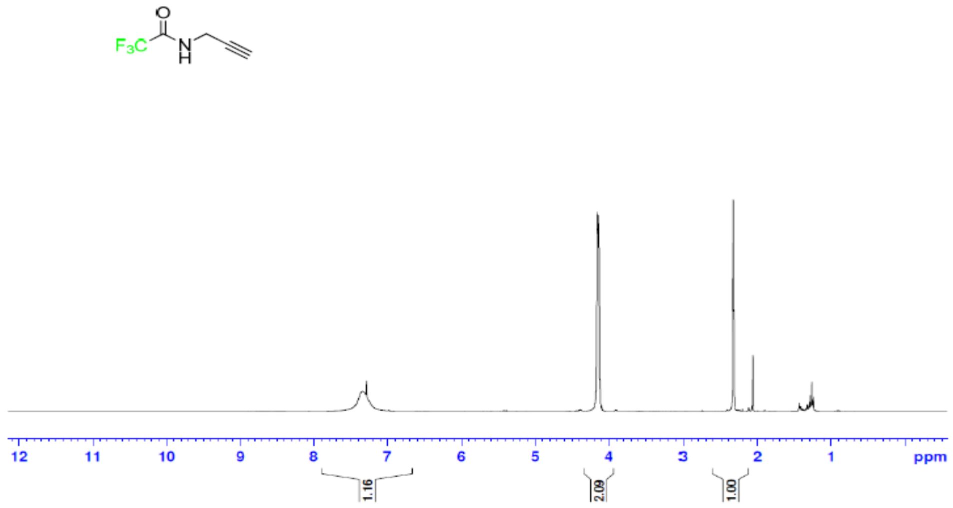

^{1}H NMR Spectrum of compound **4**

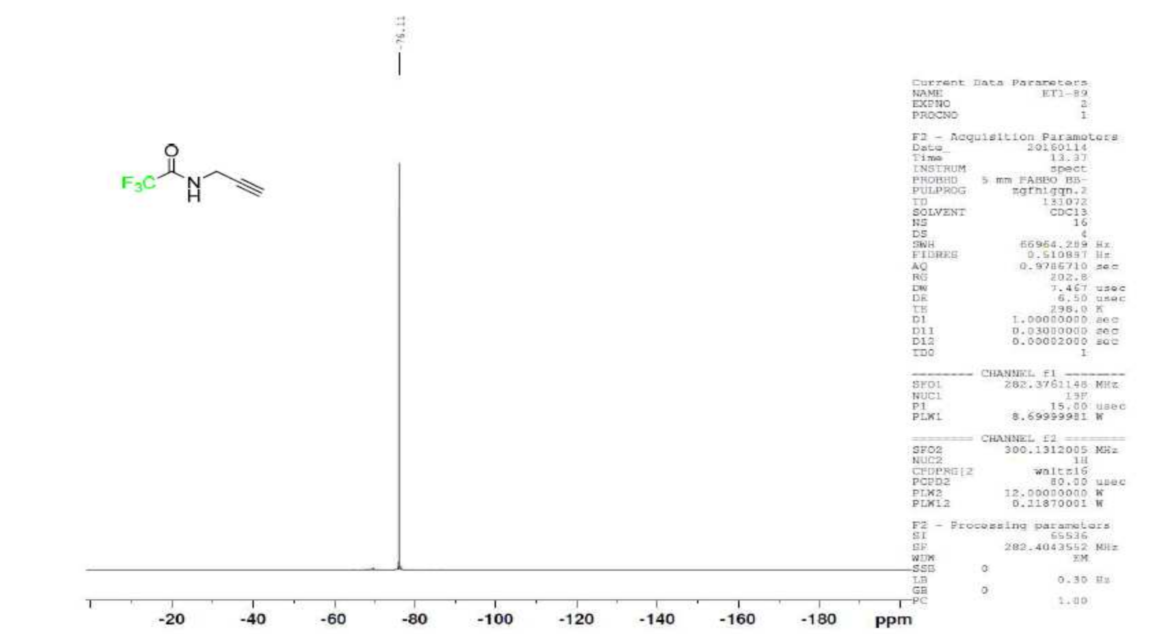

^{19}F NMR Spectrum of compound **4**

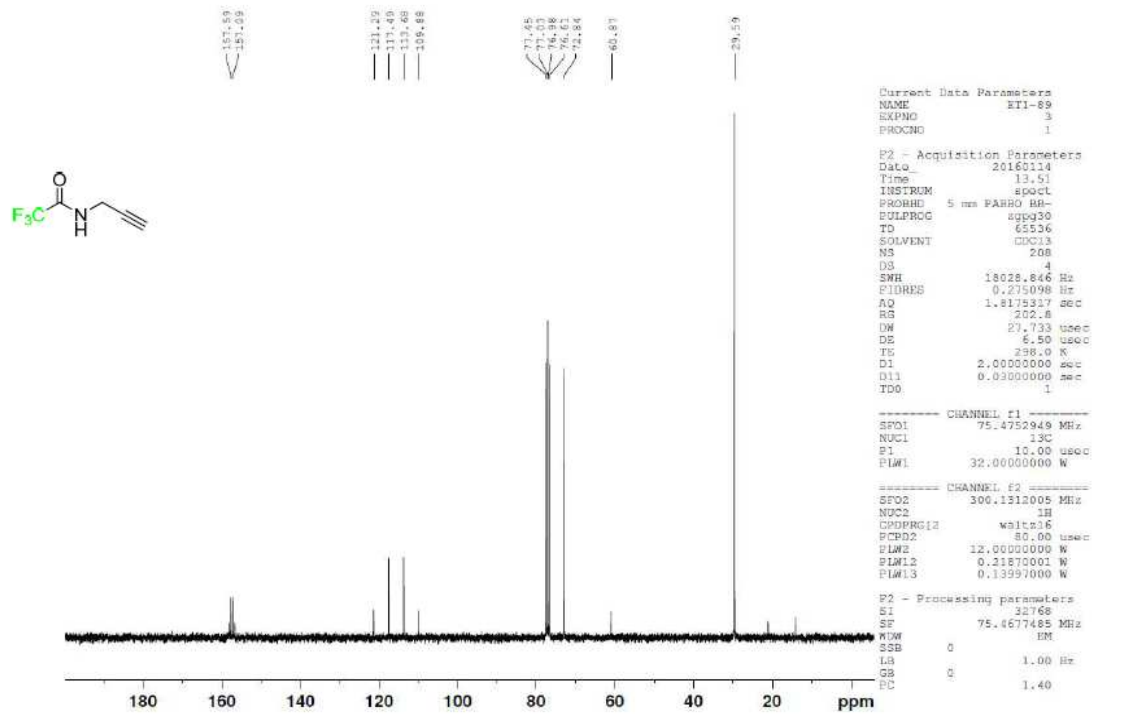

^{13}C NMR Spectrum of compound **4**

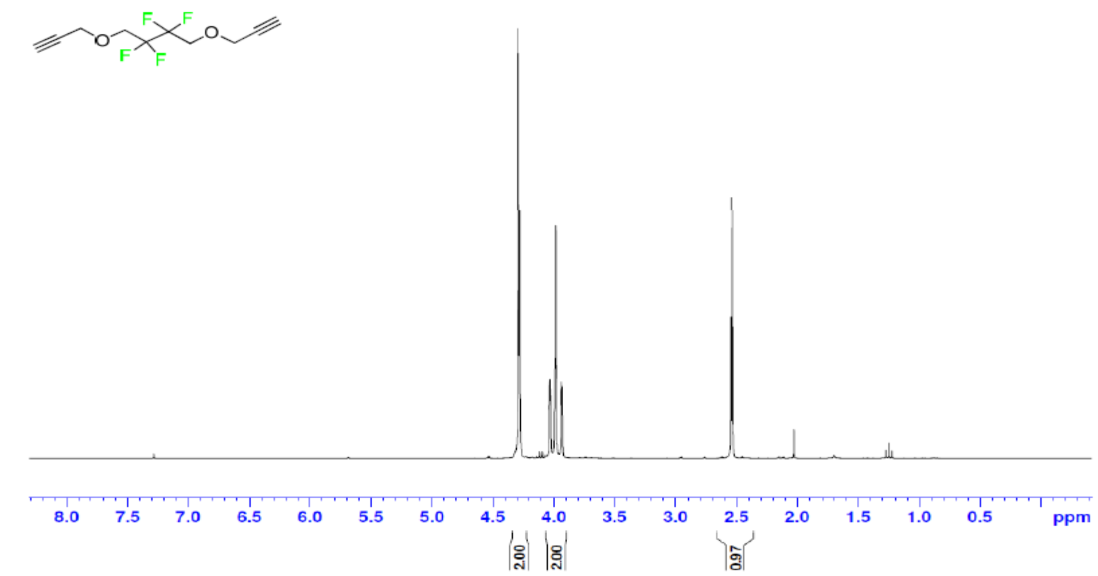

^{1}H NMR Spectrum of compound **6**

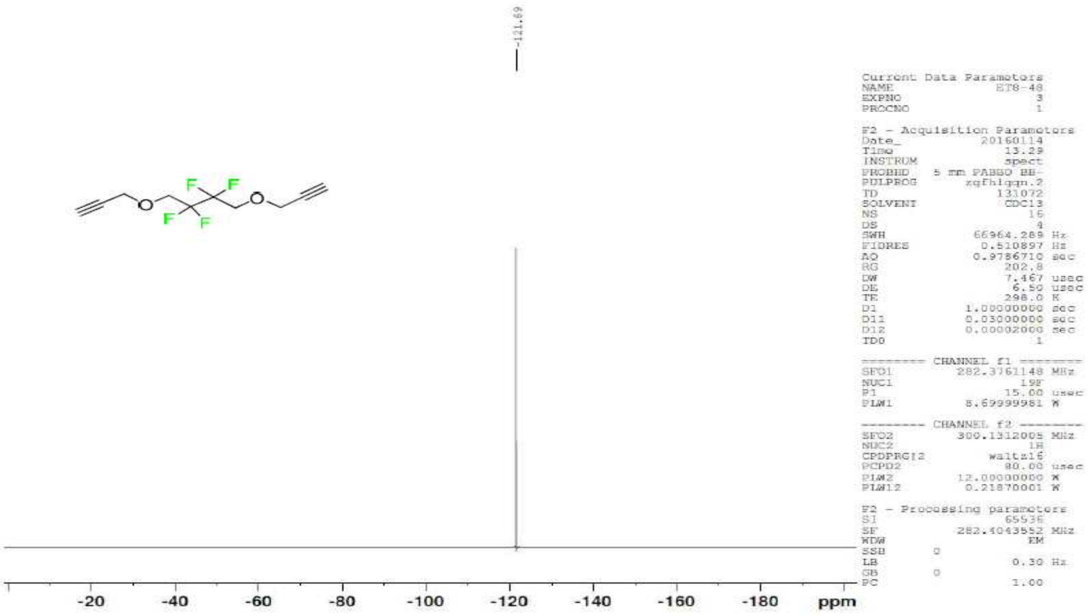

^{13}C NMR Spectrum of compound **6**

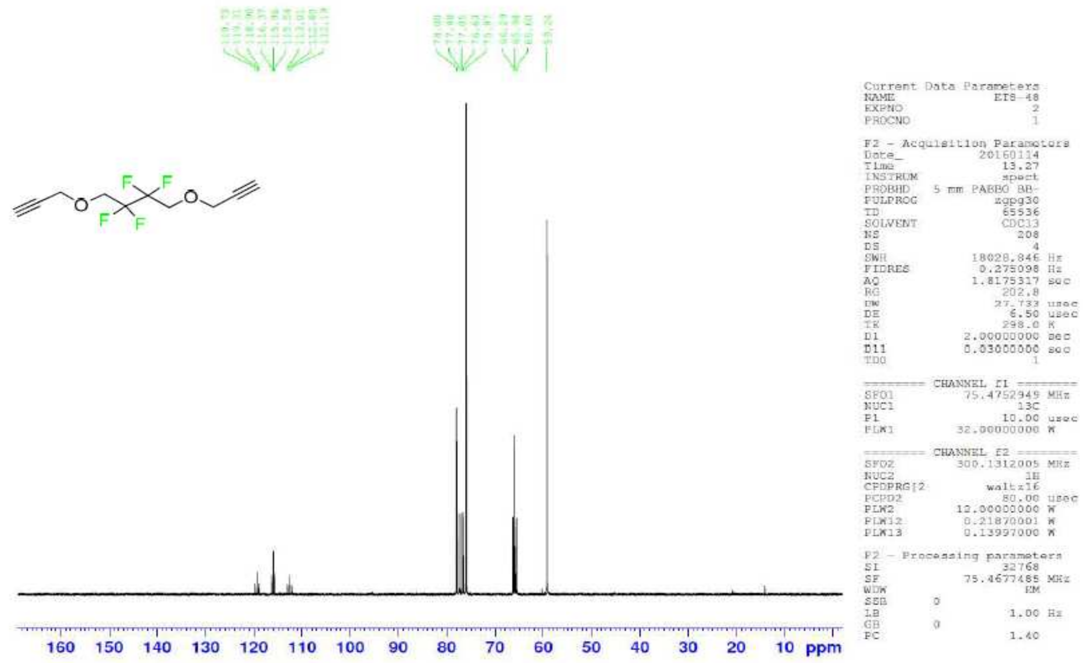

^{13}C NMR Spectrum of compound **6**

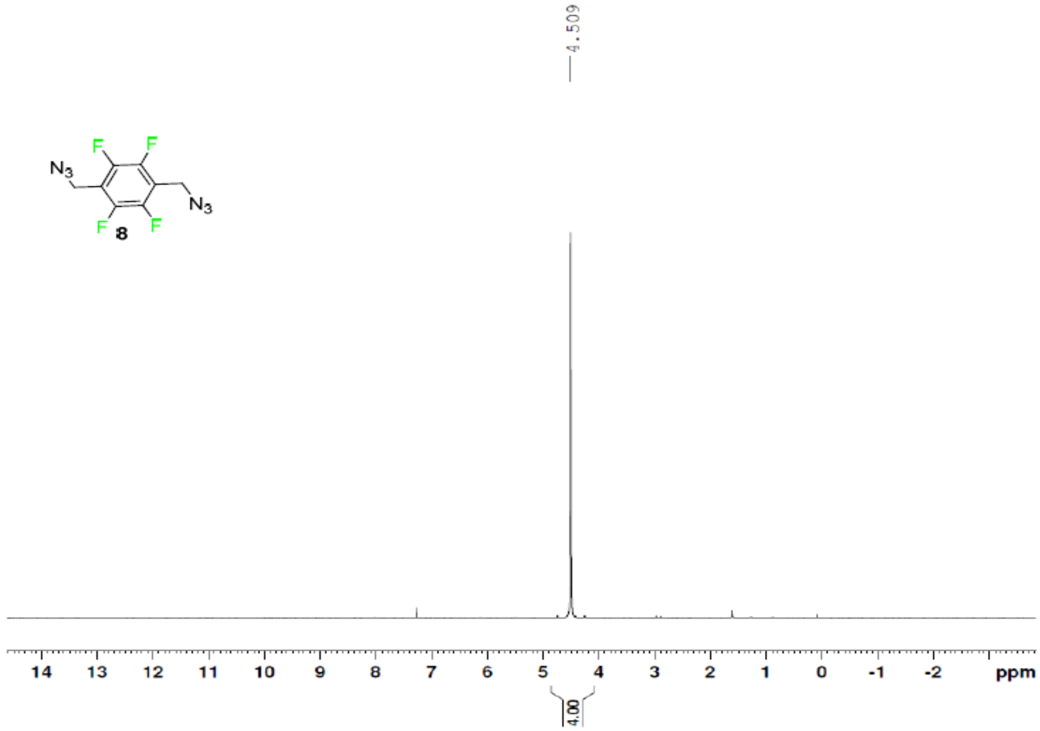

^{1}H NMR Spectrum of compound **8**

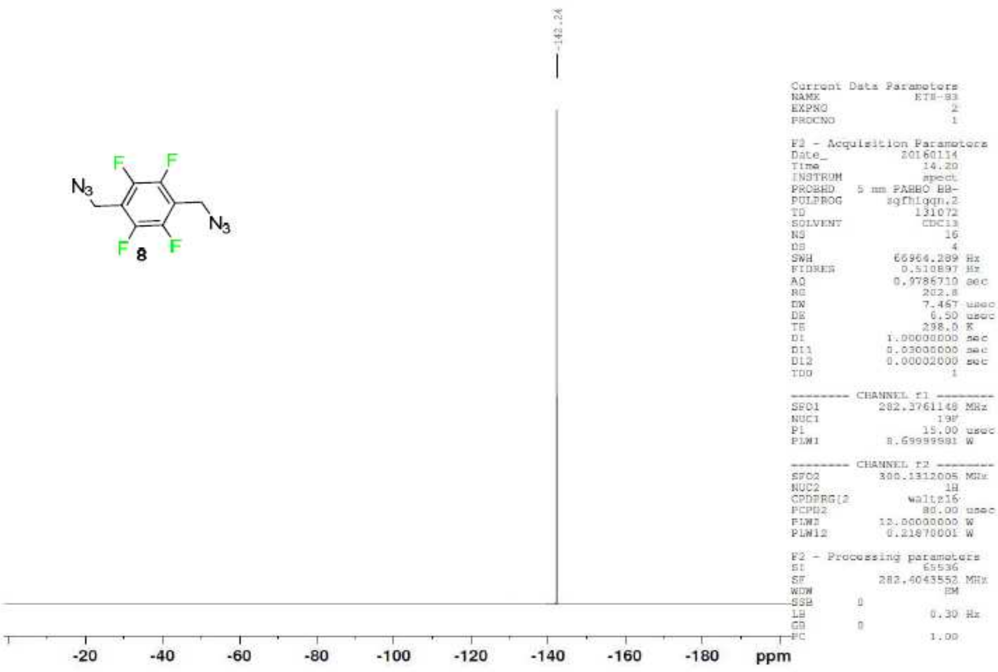

^{19}F NMR Spectrum of compound **8**

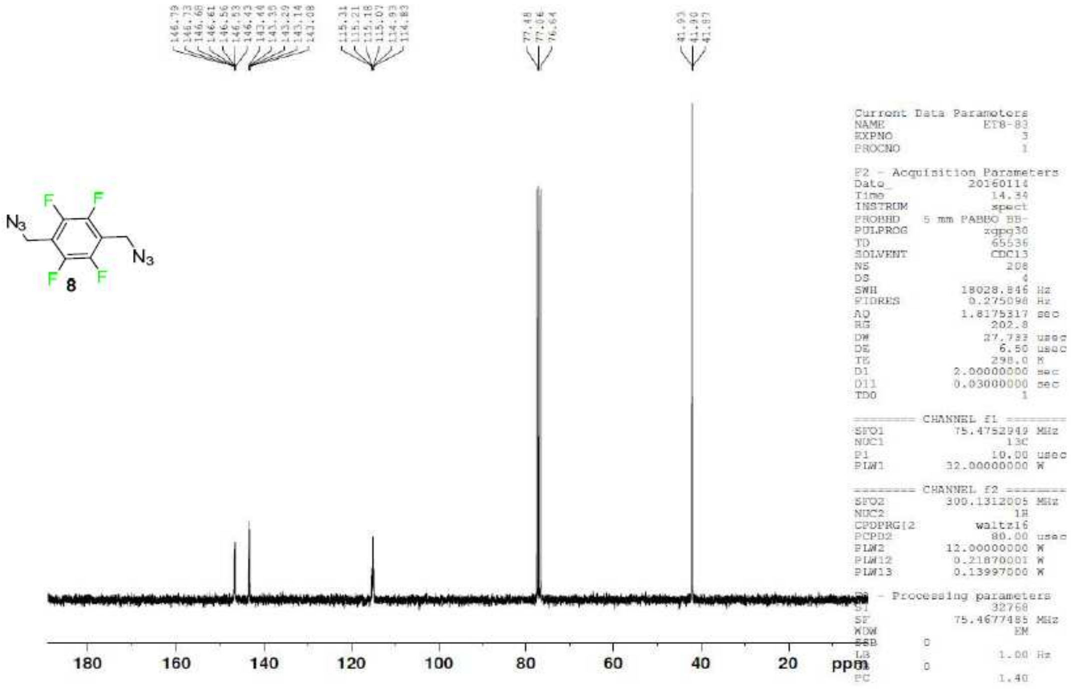

^{13}C NMR Spectrum of compound **8**

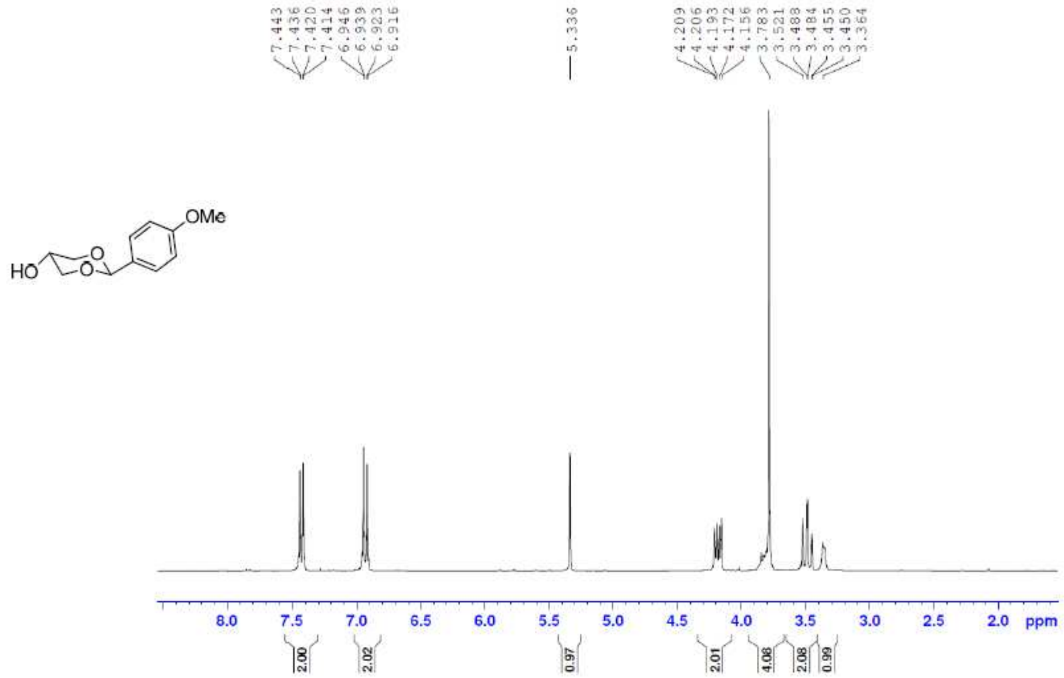

^{1}H NMR Spectrum of compound **9**

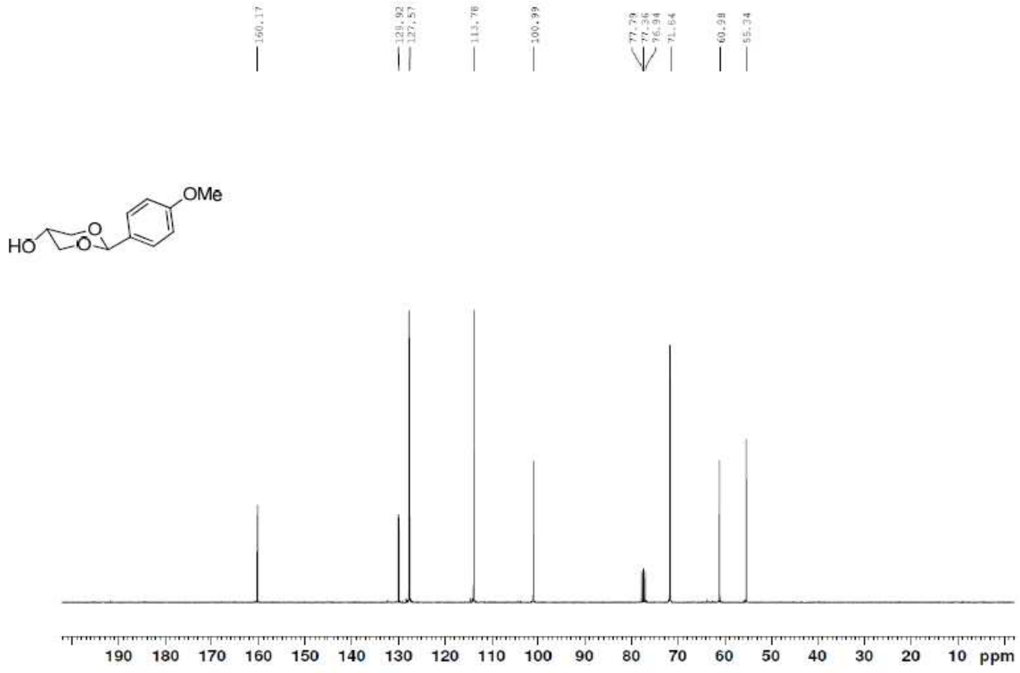

^{13}C NMR Spectrum of compound **9**

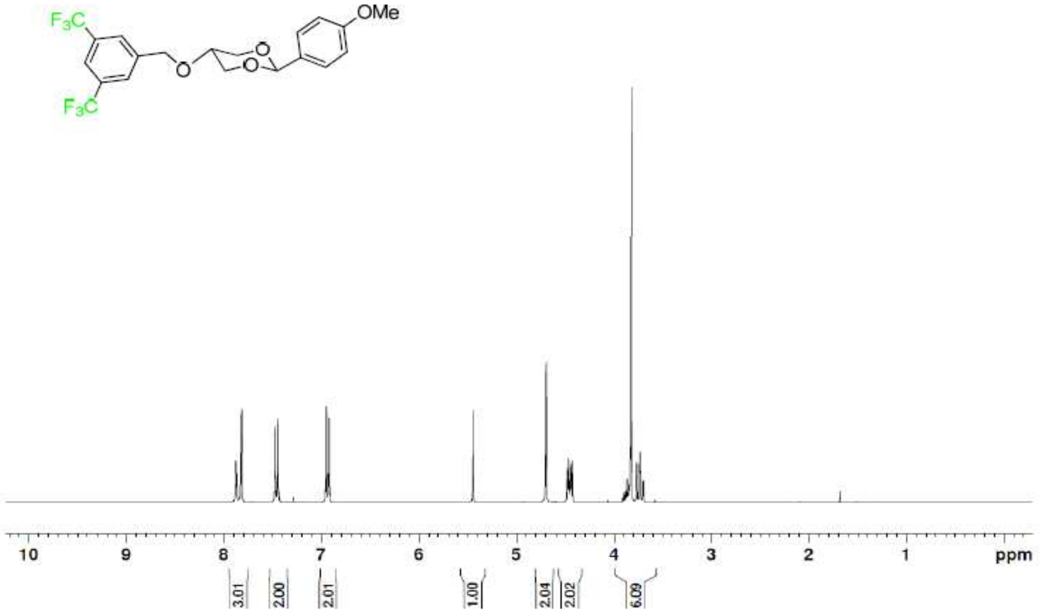

^{1}H NMR Spectrum of compound **10**

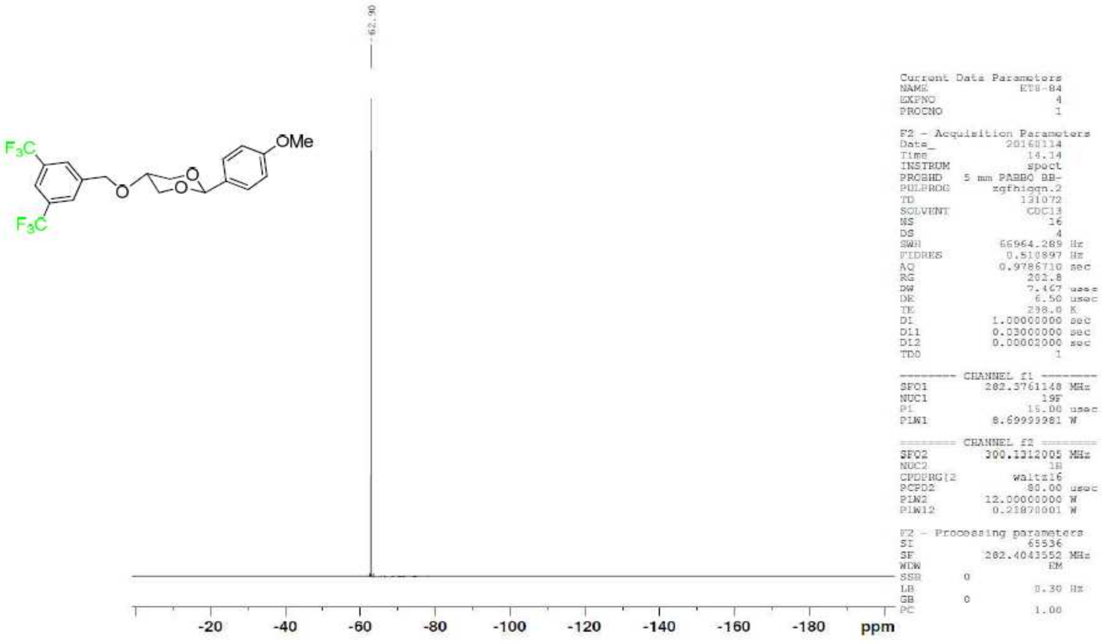

^{19}F NMR Spectrum of compound **10**

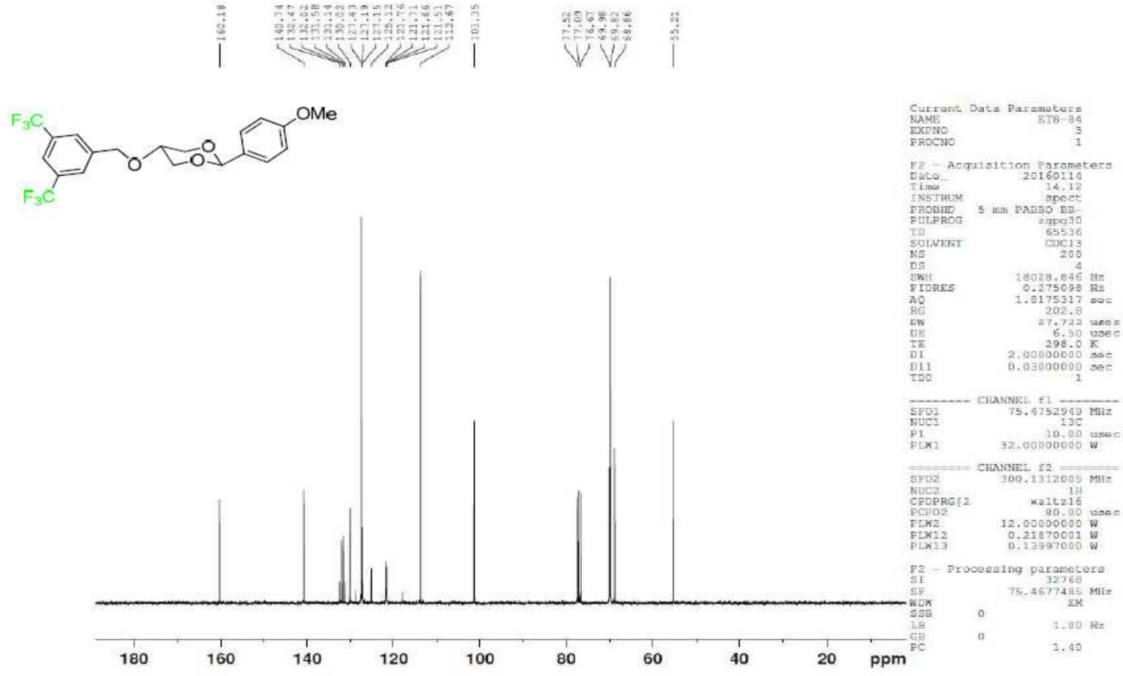

^{13}C NMR Spectrum of compound **10**

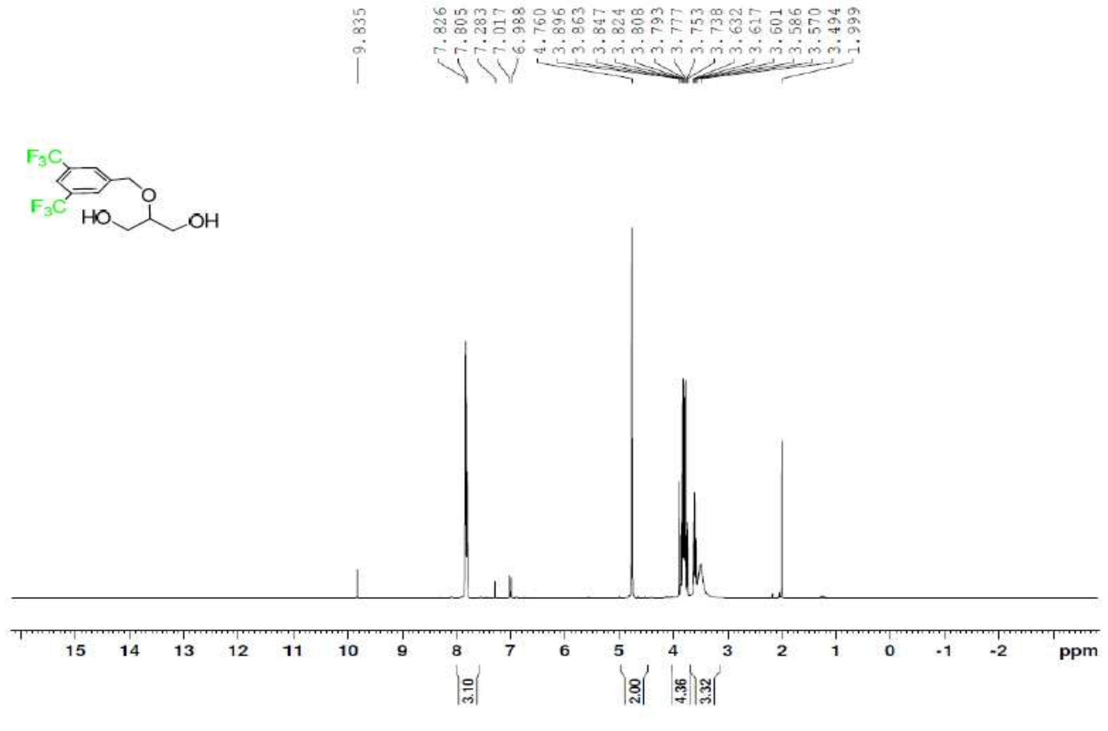

^{1}H NMR Spectrum of compound **11**

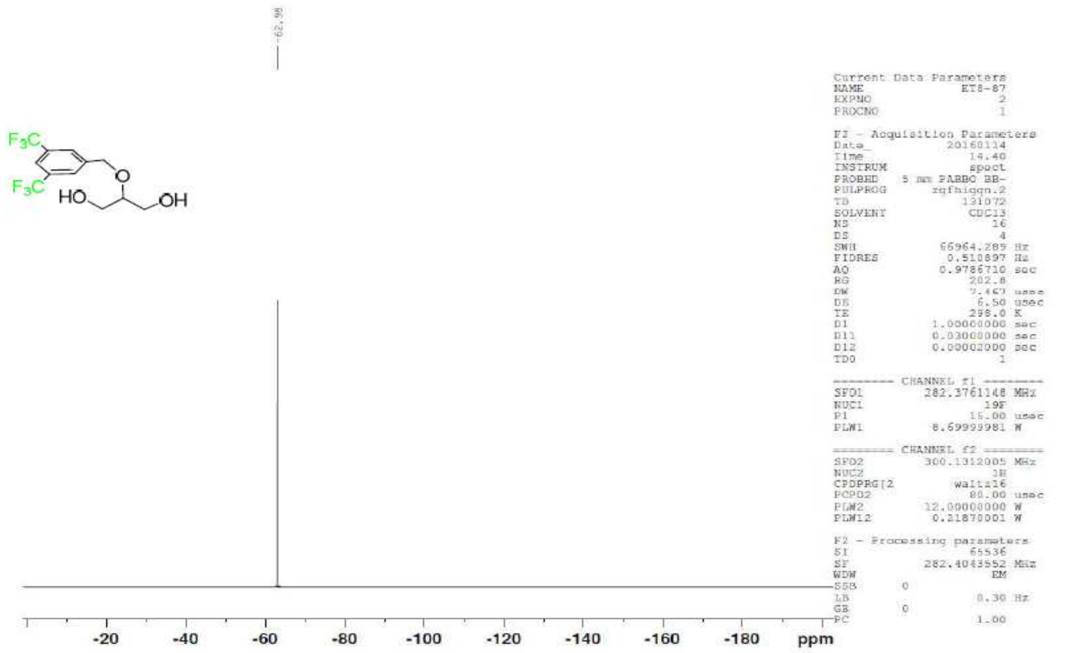

^{19}F NMR Spectrum of compound **11**

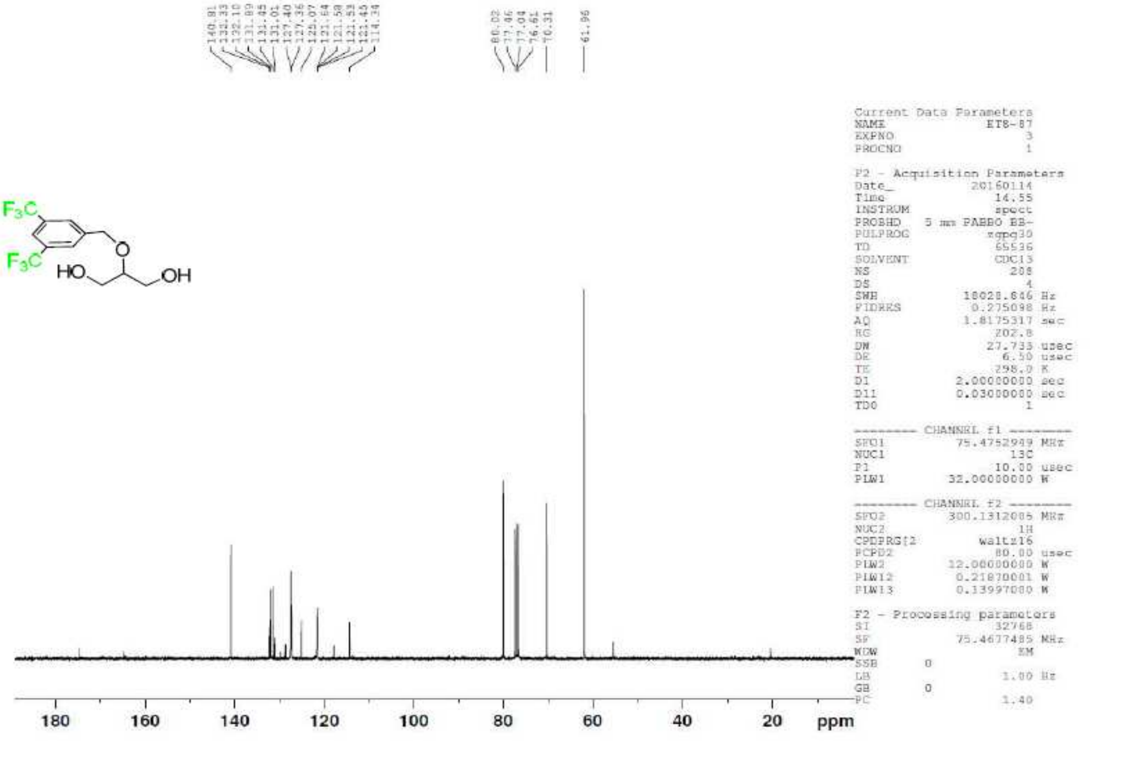

^{13}C NMR Spectrum of compound **11**

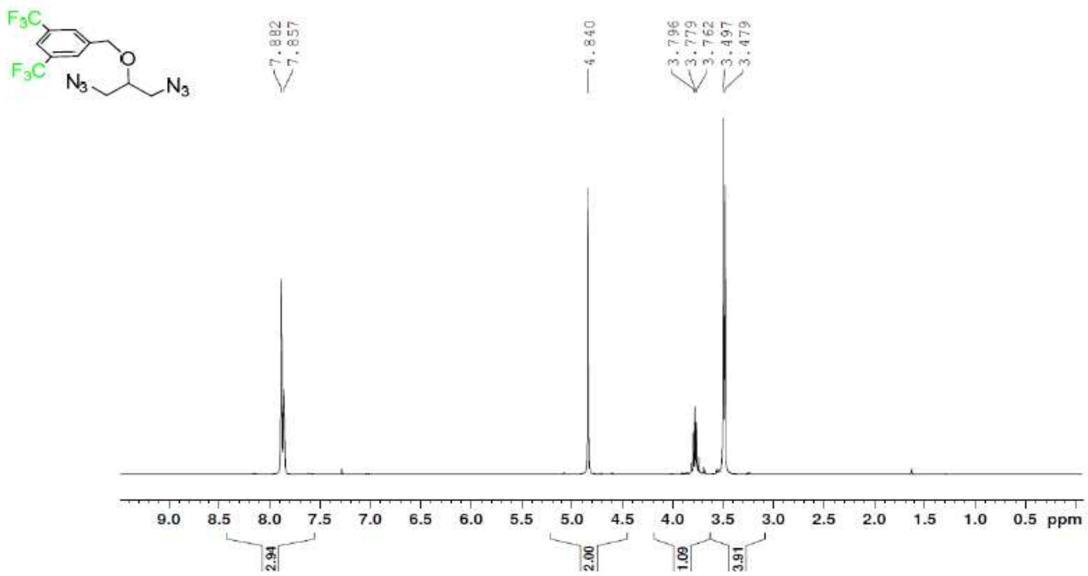

^{1}H NMR Spectrum of compound **12**

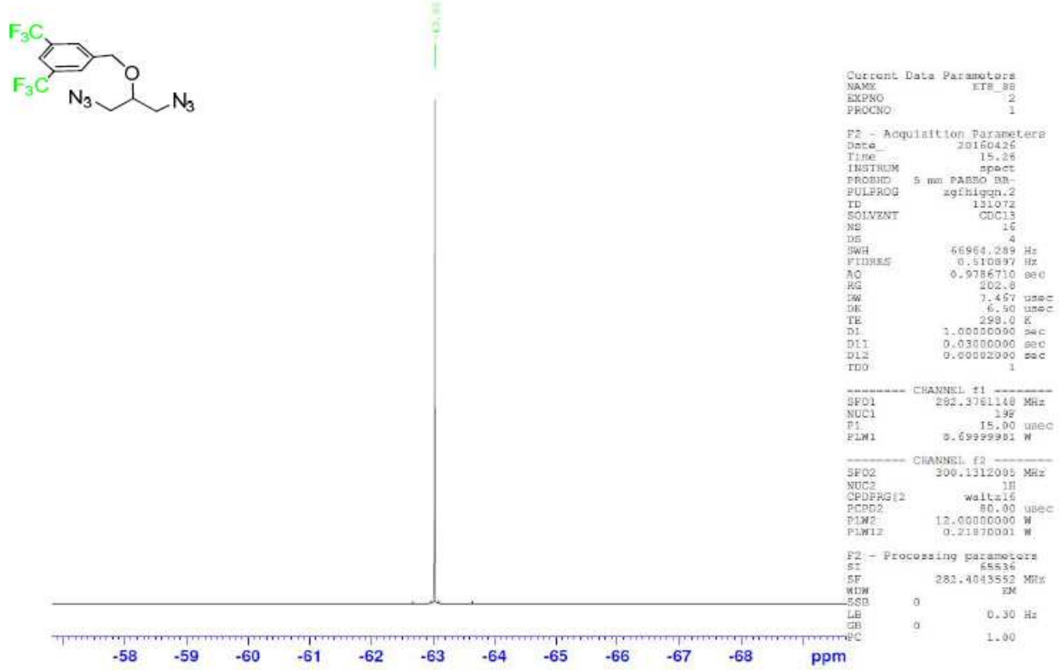

^{19}F NMR Spectrum of compound **12**

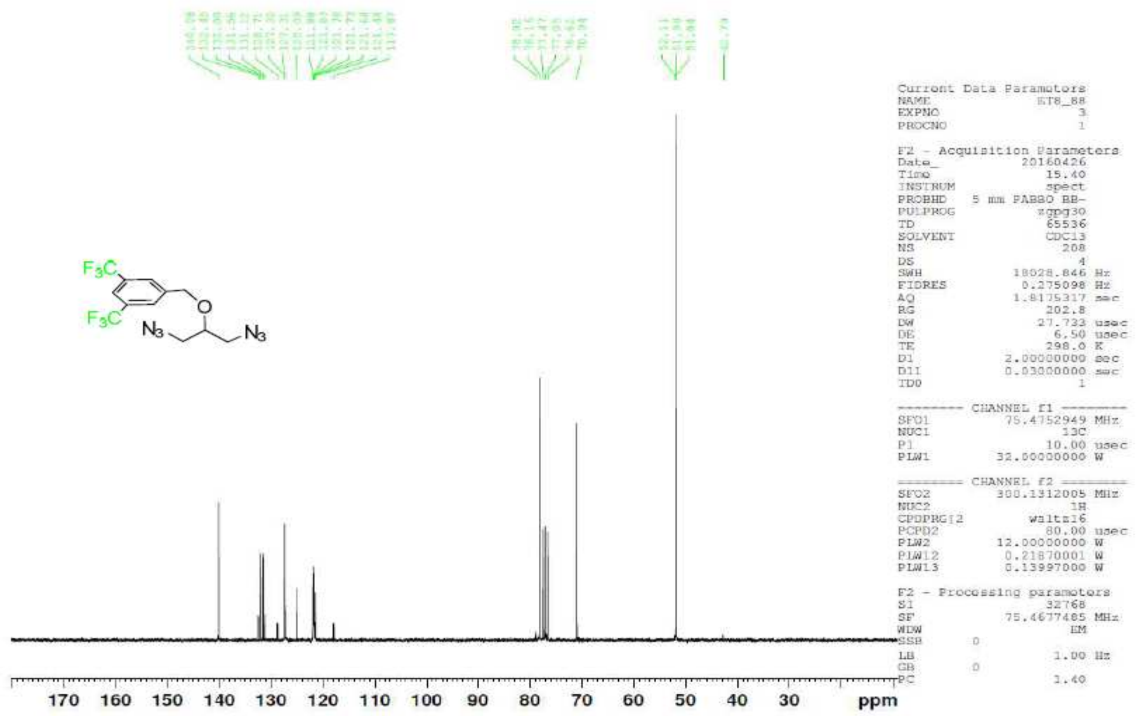

^{13}C NMR Spectrum of compound **12**

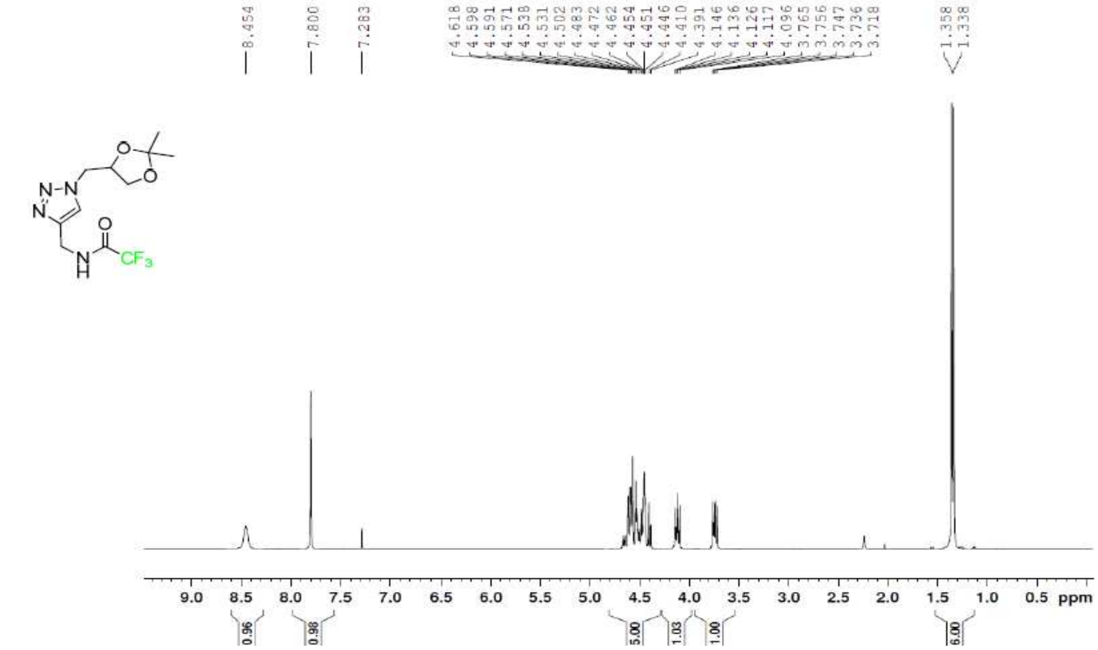

^{1}H NMR Spectrum of compound **13**

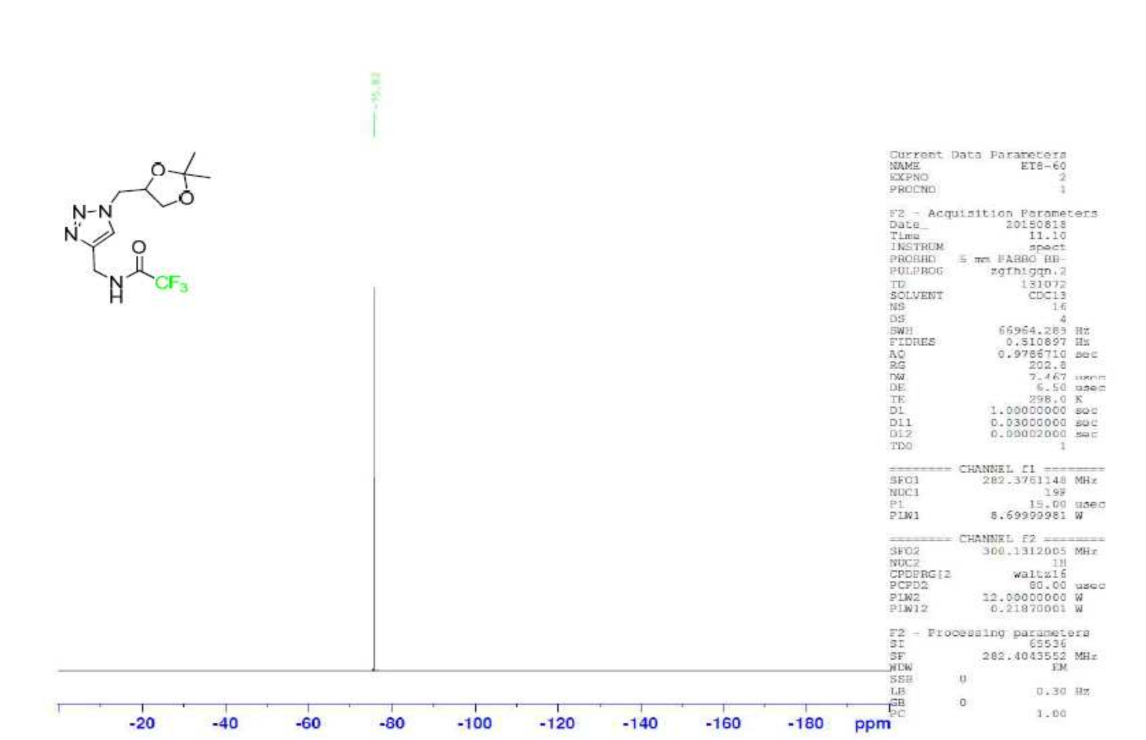

^{19}F NMR Spectrum of compound **13**

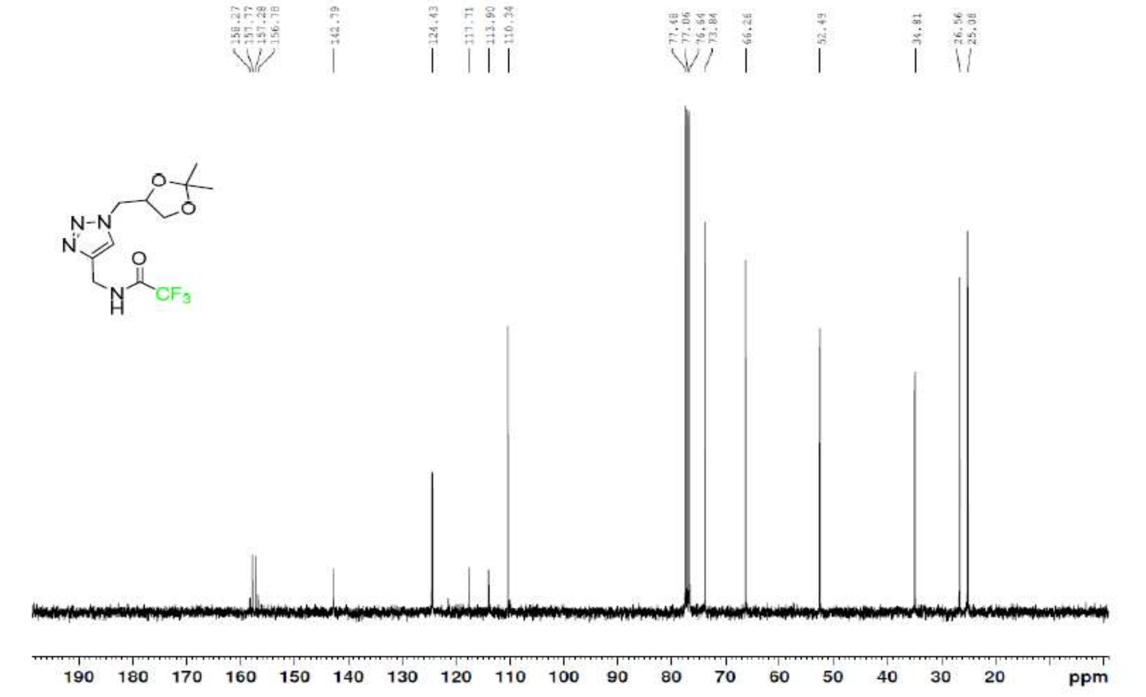

^{13}C NMR Spectrum of compound **13**

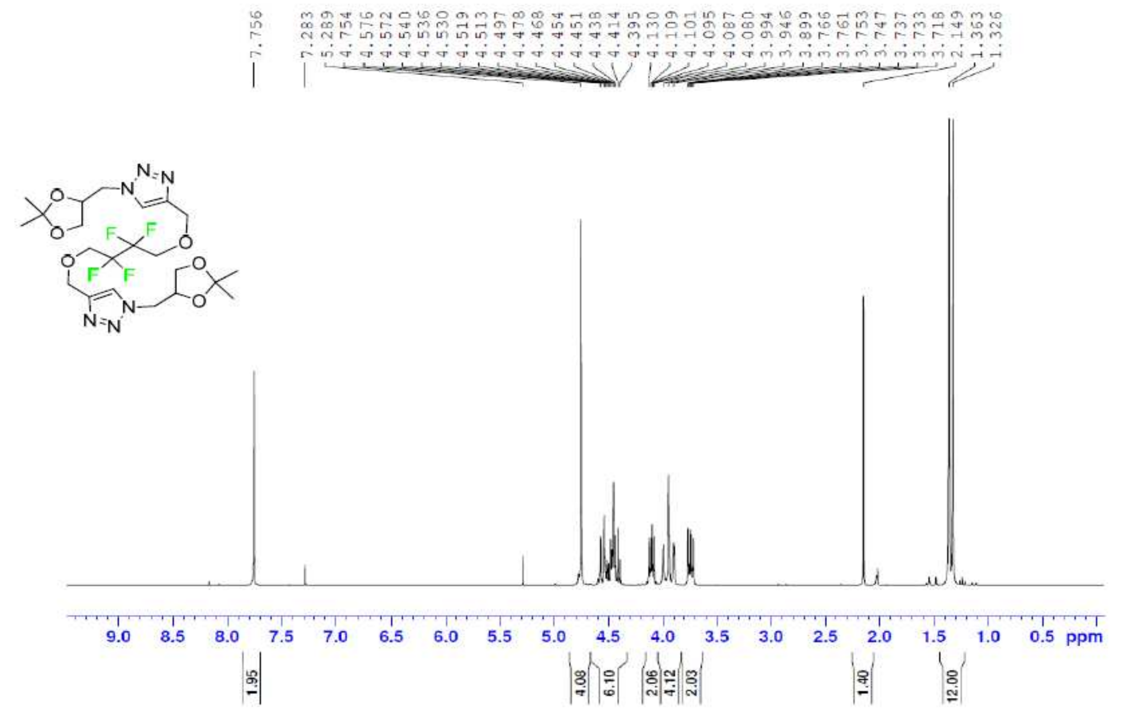

^{1}H NMR Spectrum of compound **14**

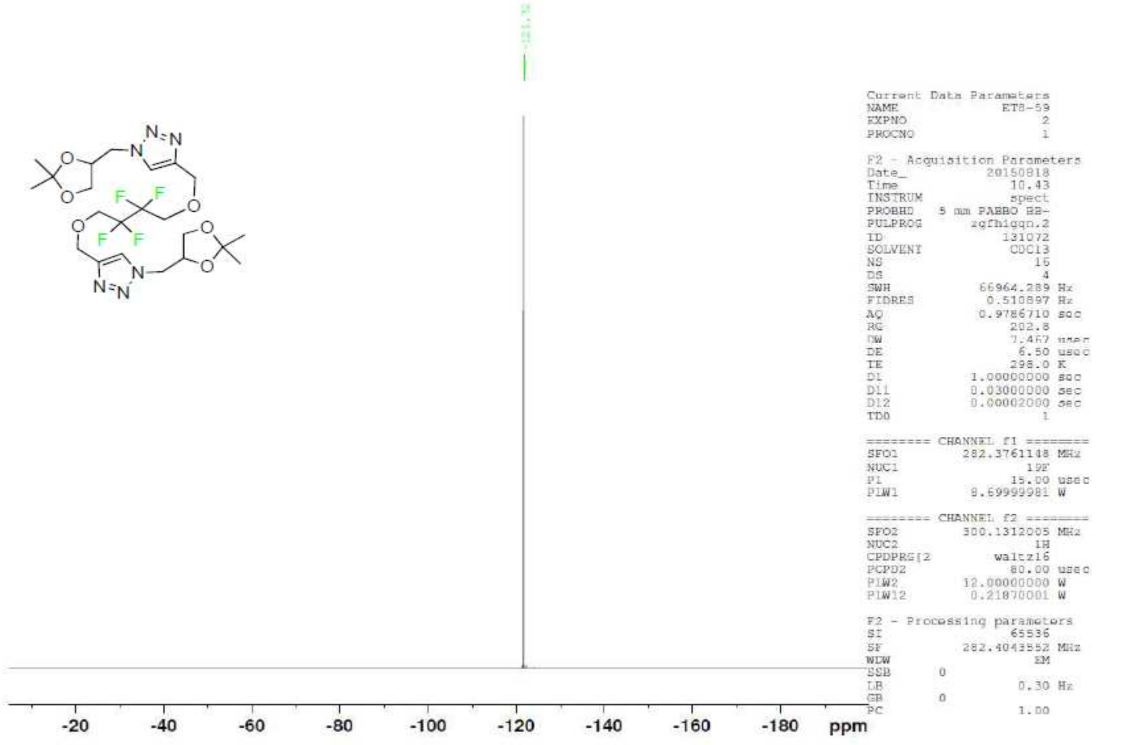

^{19}F NMR Spectrum of compound **14**

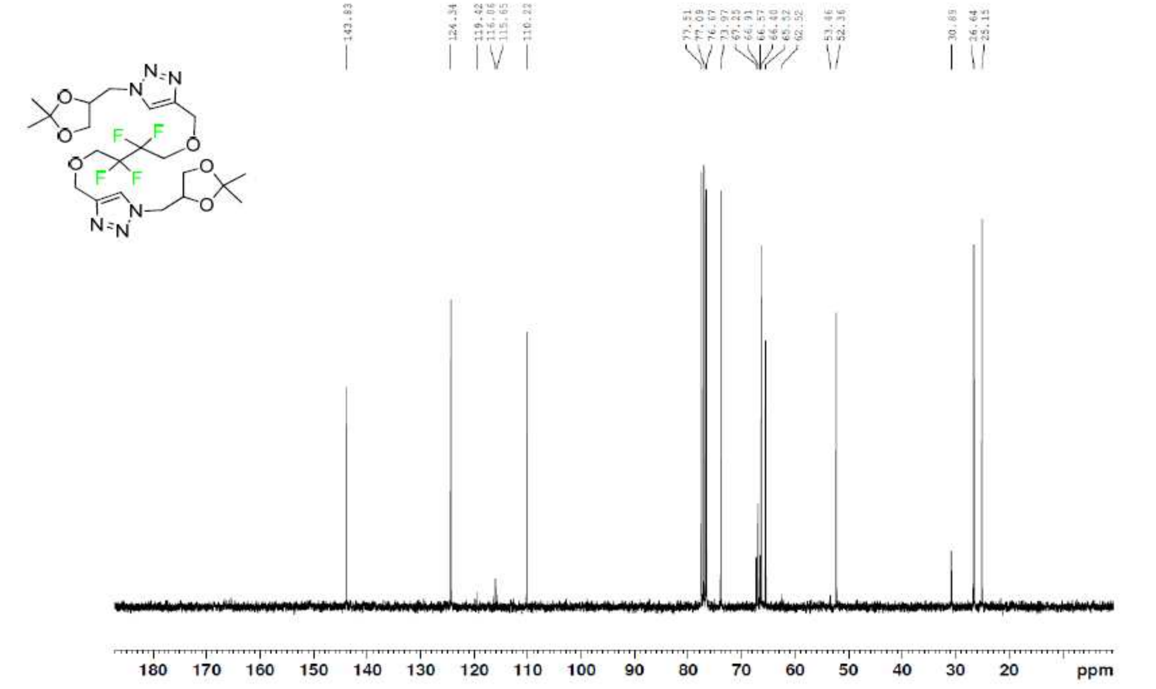

^{13}C NMR Spectrum of compound **14**

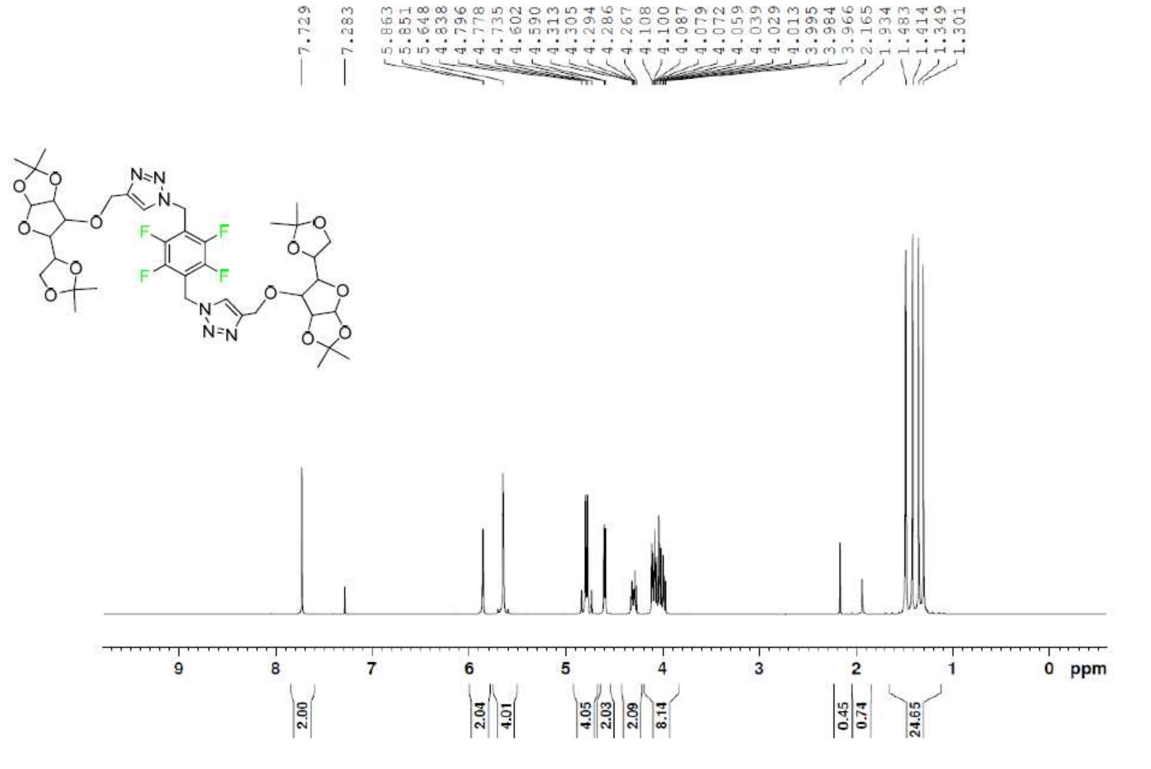

^{1}H NMR Spectrum of compound **15**

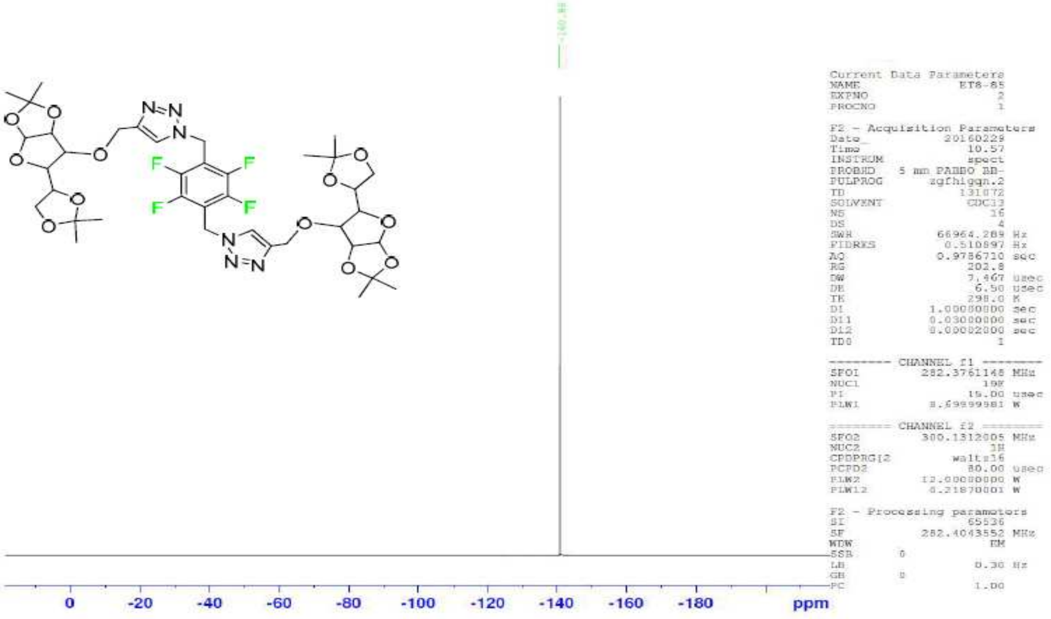

^{19}F NMR Spectrum of compound **15**

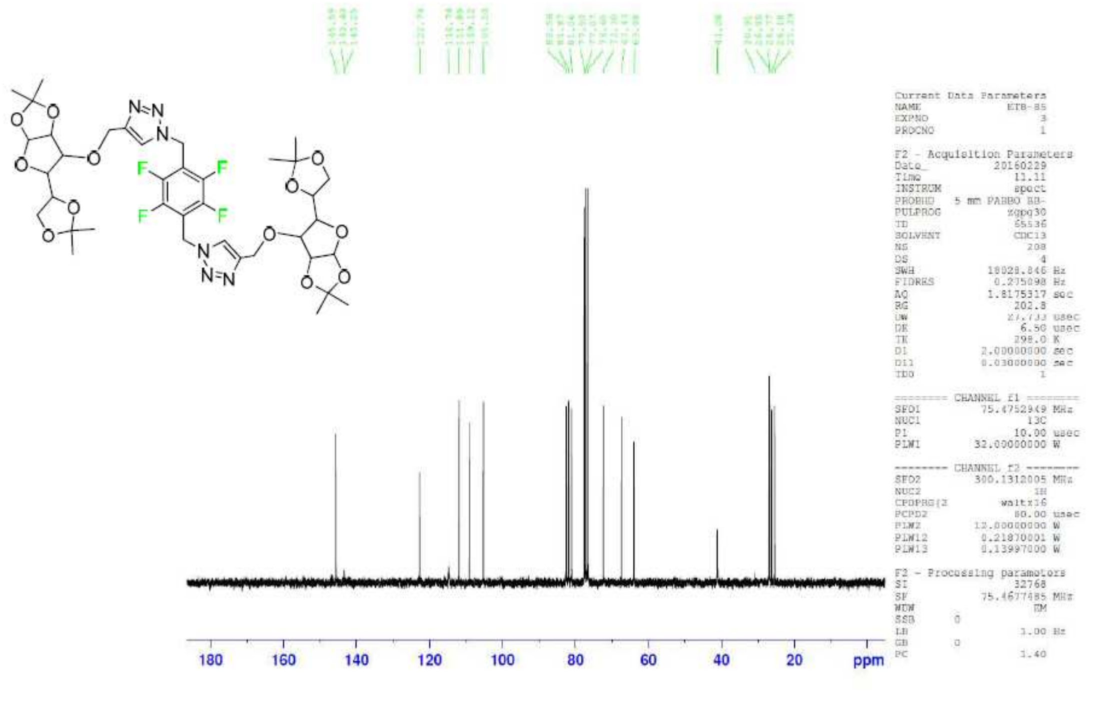

^{13}C NMR Spectrum of compound **15**

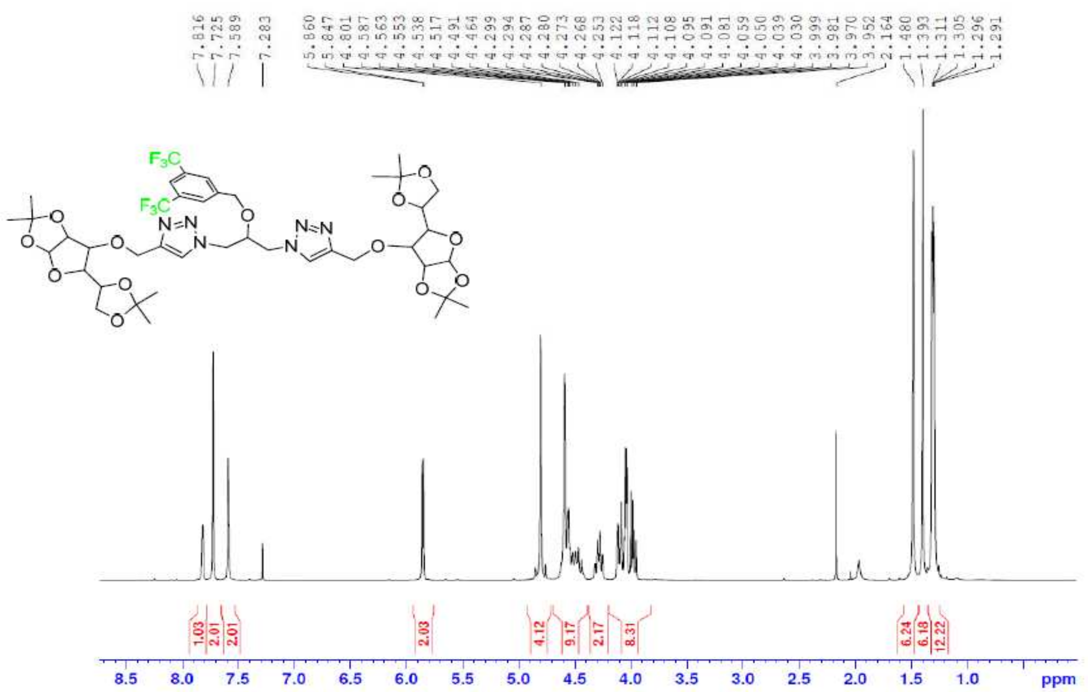

^{1}H NMR Spectrum of compound **16**

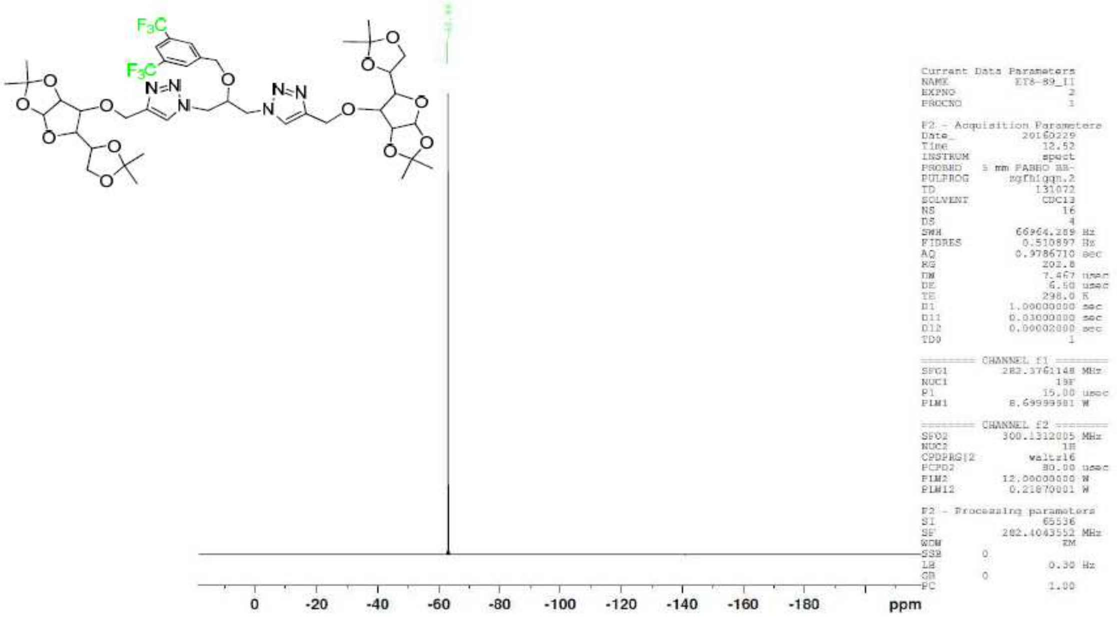

^{19}F NMR Spectrum of compound **16**

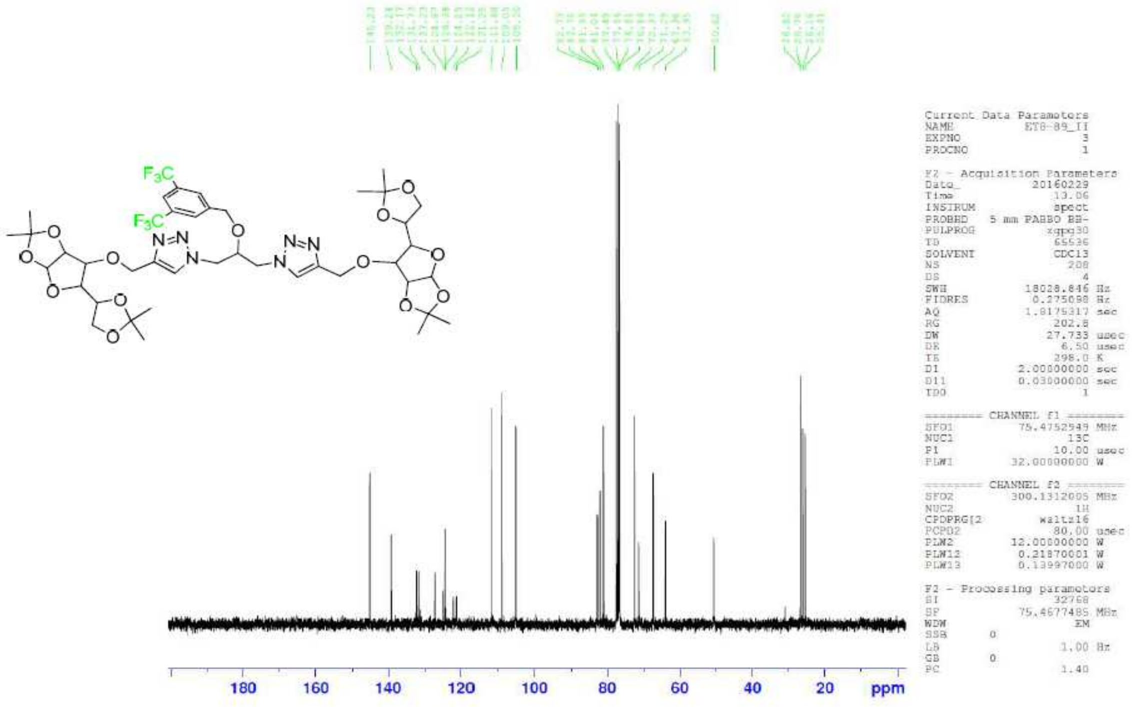

^{13}C NMR Spectrum of compound **16**

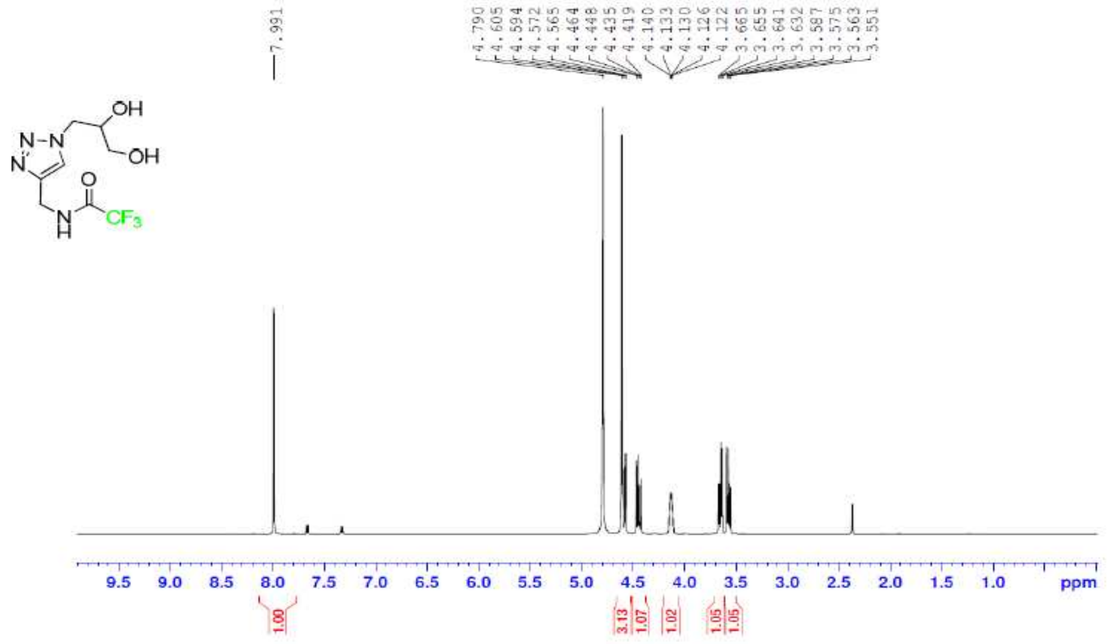

^{1}H NMR Spectrum of compound **ET0863**

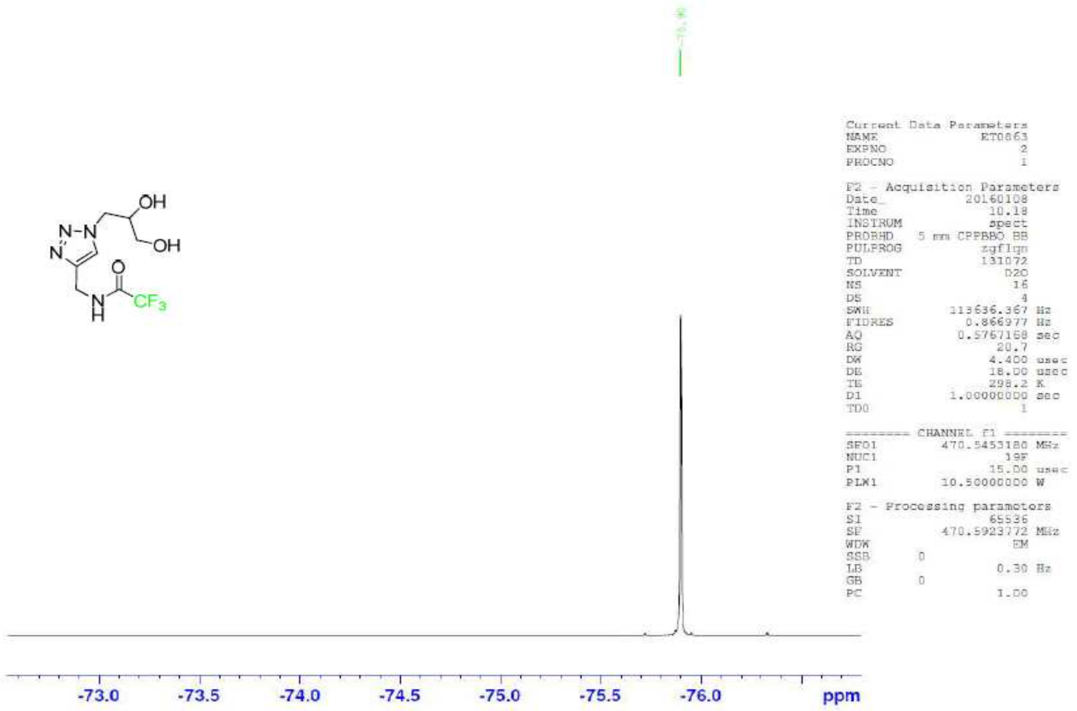

^{19}F NMR Spectrum of compound **ET0863**

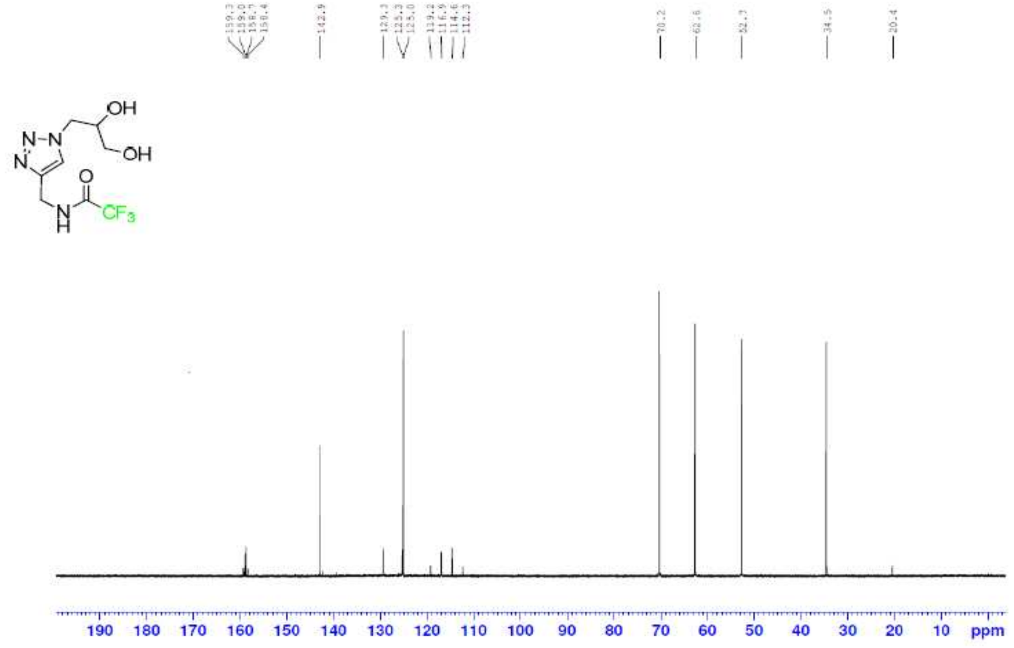

^{13}C NMR Spectrum of compound **ET0863**

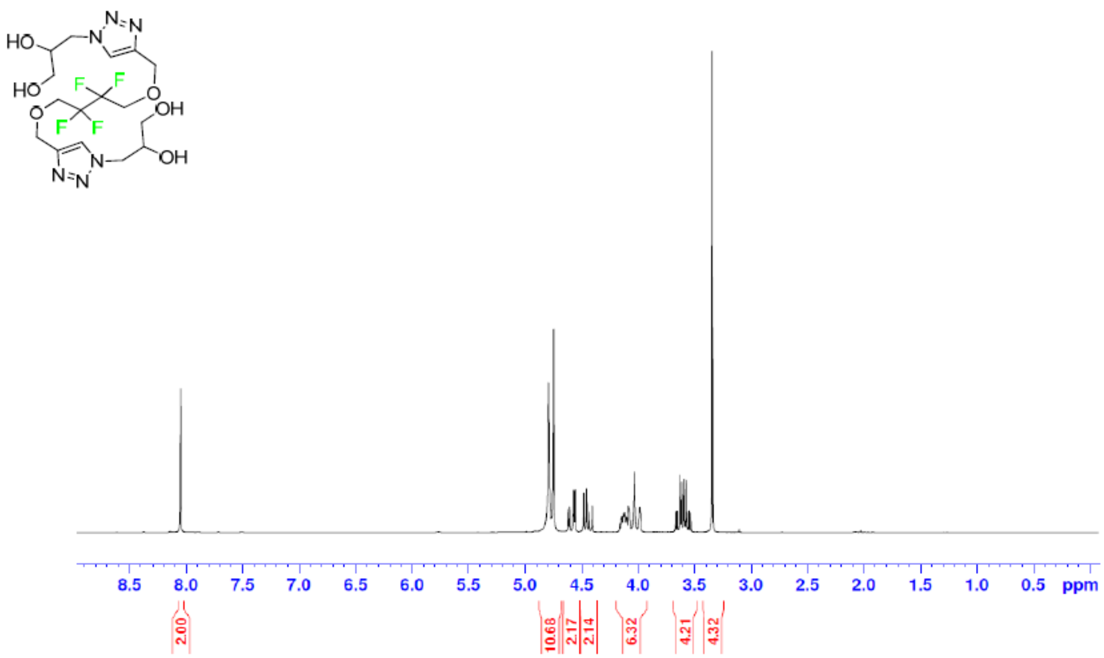

^{1}H NMR Spectrum of compound **ET0876**

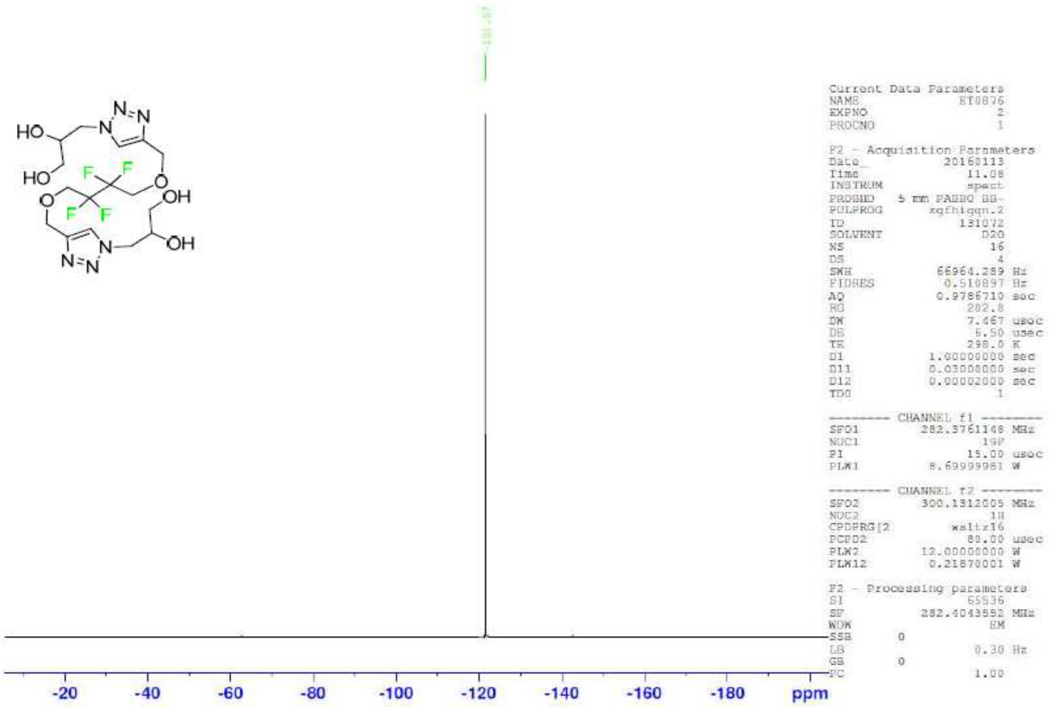

^{19}F NMR Spectrum of compound **ET0876**

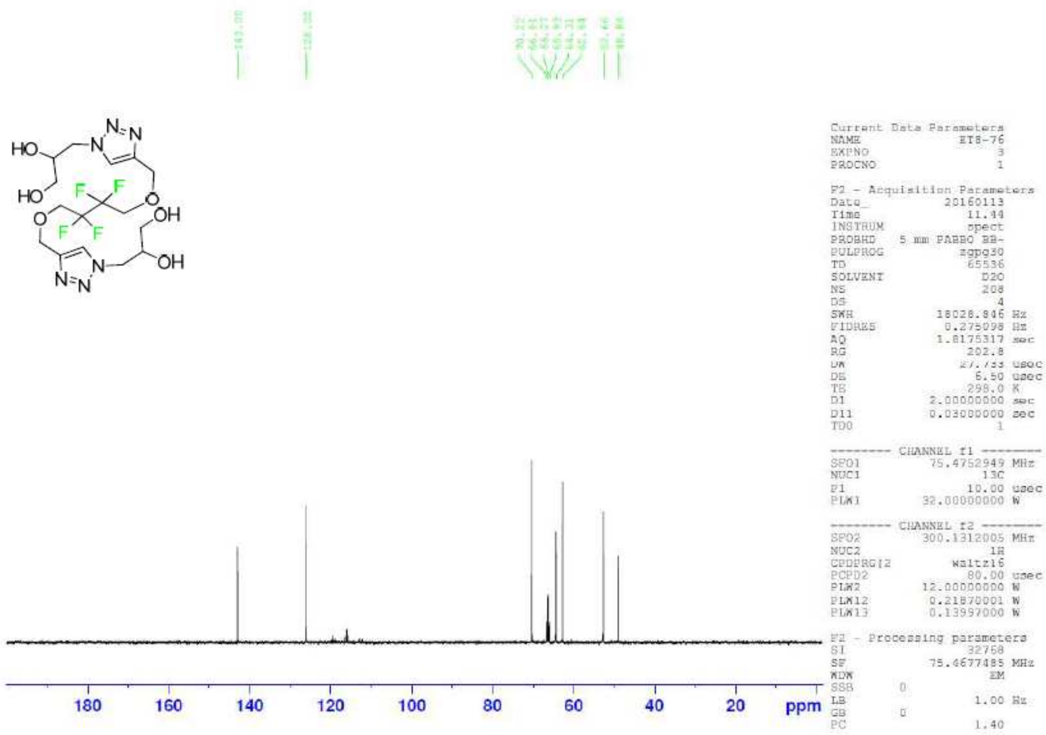

^{13}C NMR Spectrum of compound **ET0876**

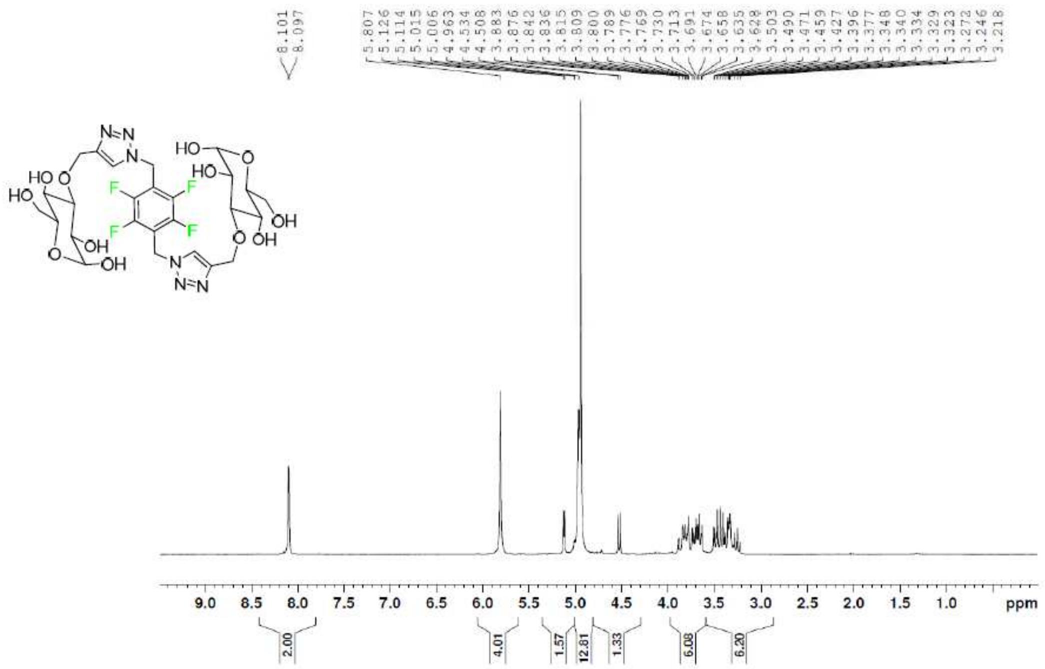

^{1}H NMR Spectrum of compound **ET0886**

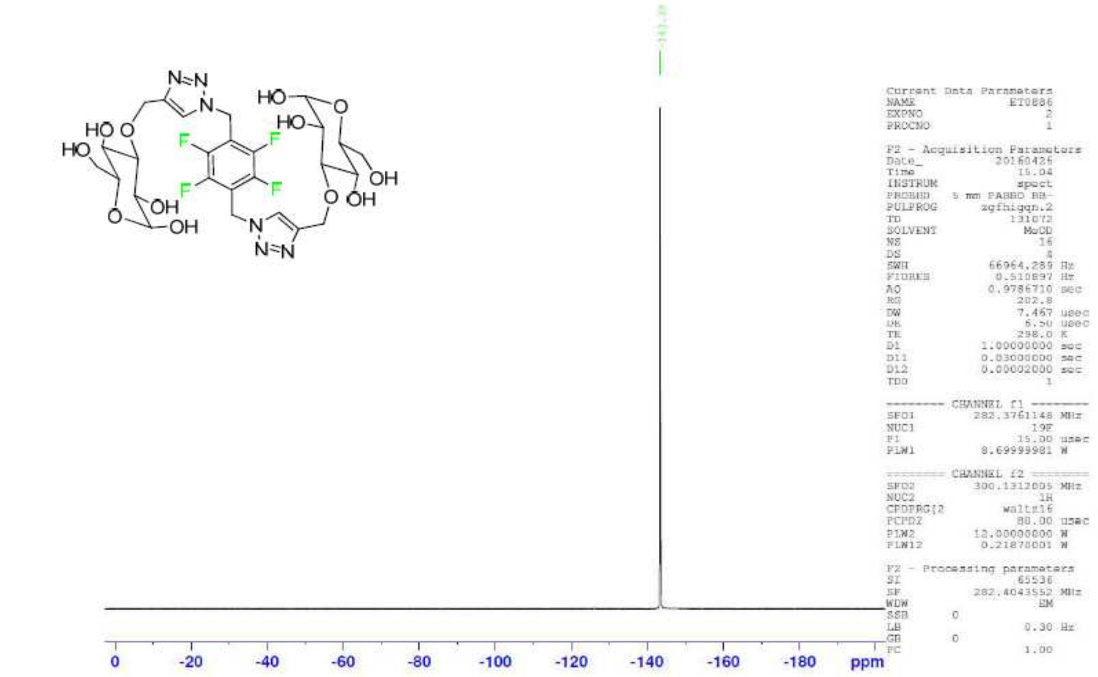

^{19}F NMR Spectrum of compound **ET0886**

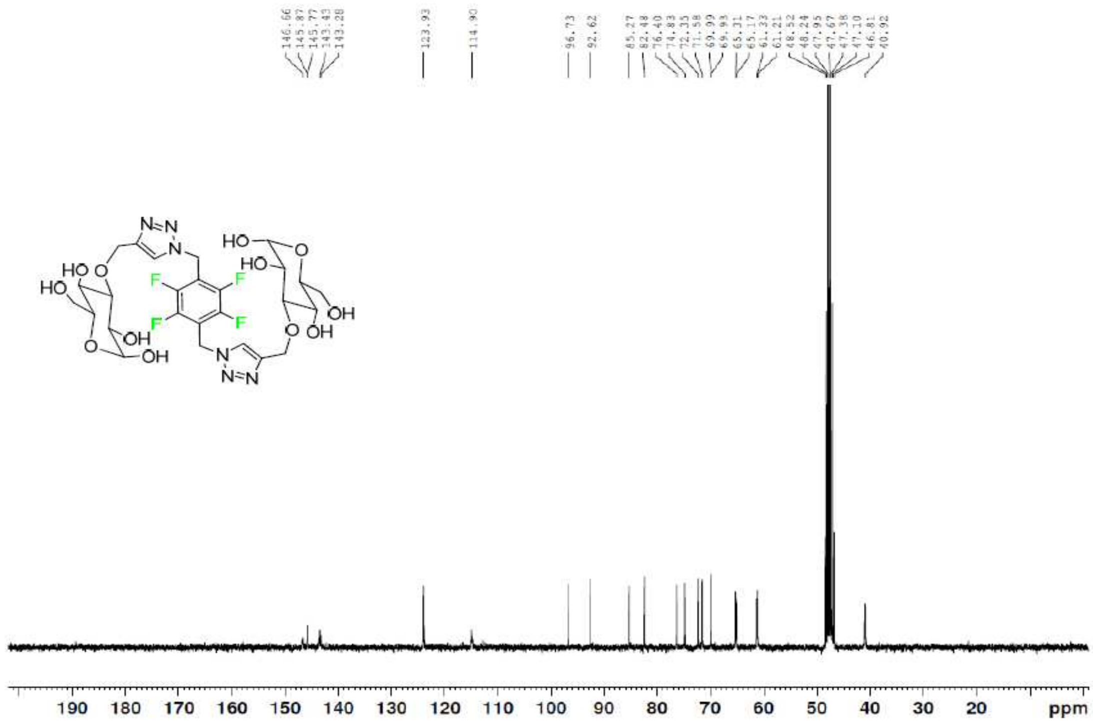

^{13}C NMR Spectrum of compound **ET0886**

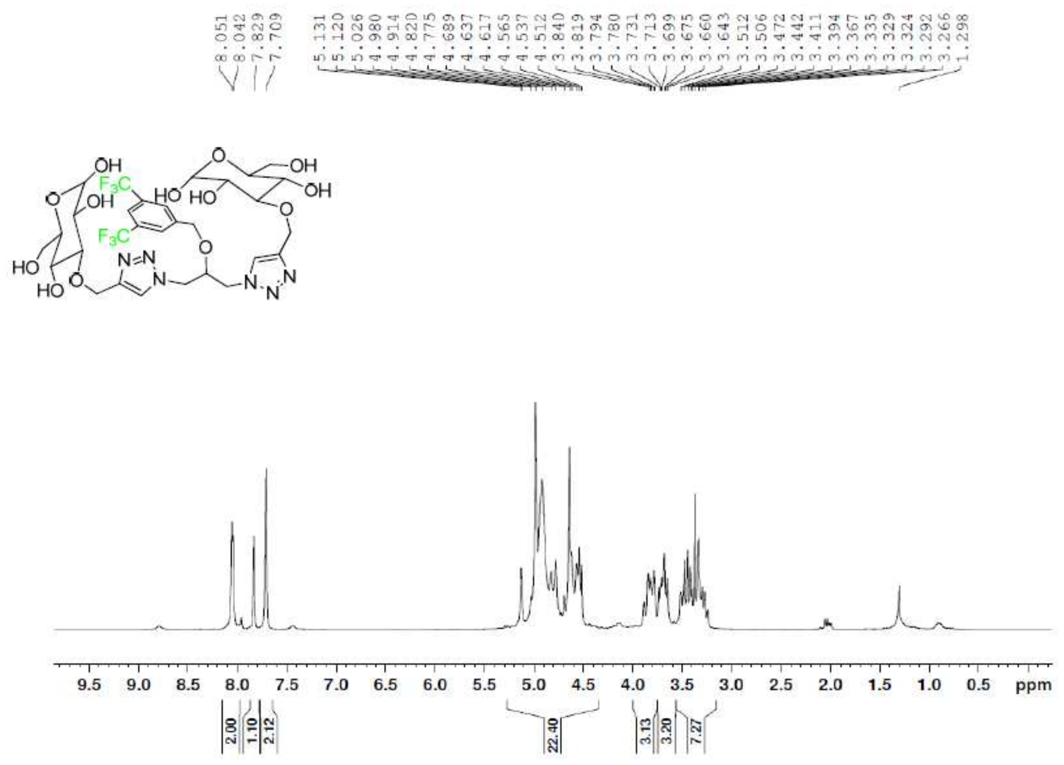

^{1}H NMR Spectrum of compound **ET0890**

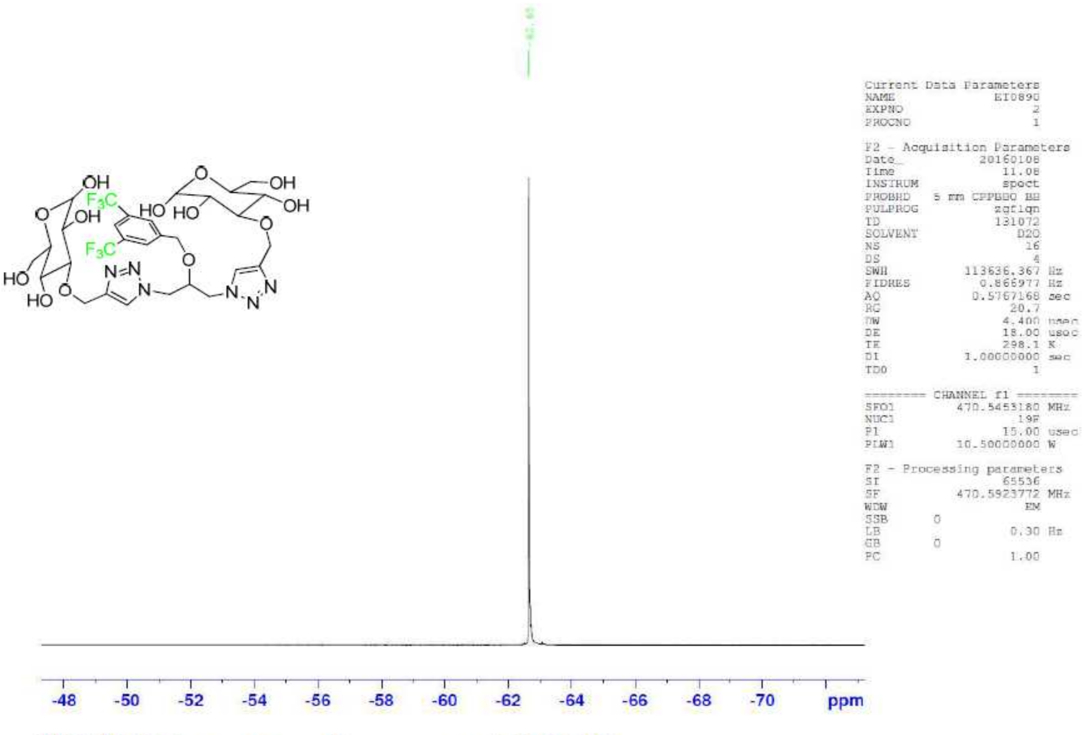

^{19}F NMR Spectrum of compound **ET0890**

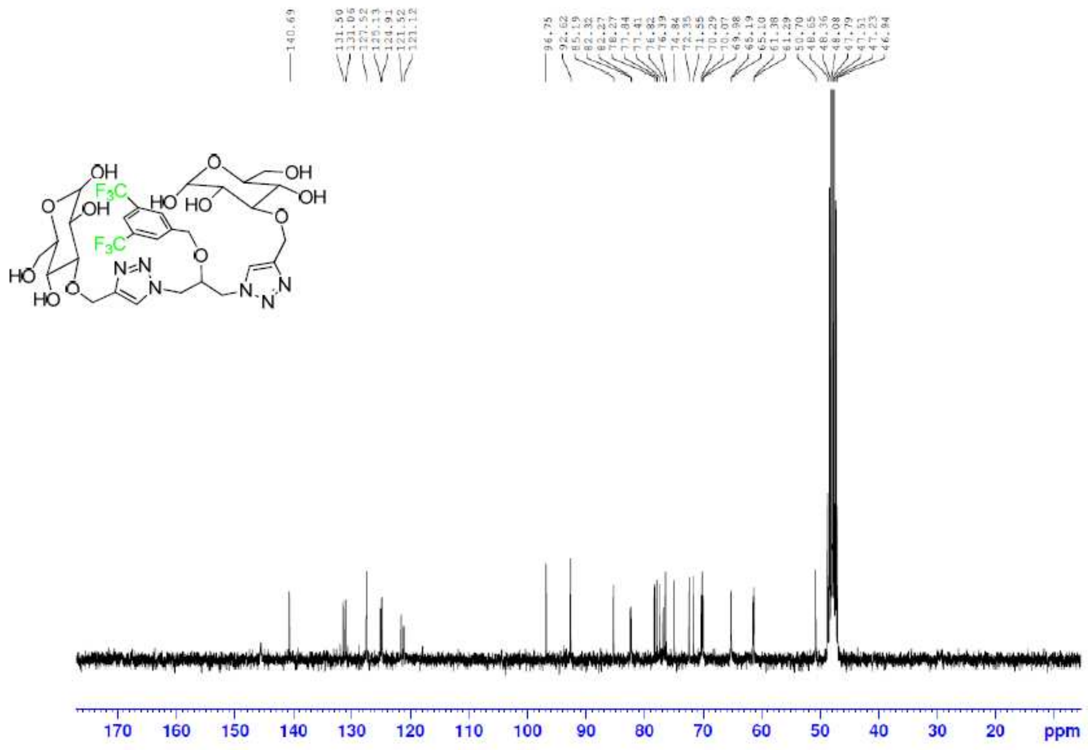

^{13}C NMR Spectrum of compound **ET0890**
